# Soft Sensor Design via Switching Observers

**DOI:** 10.3390/s23042114

**Published:** 2023-02-13

**Authors:** Fotis N. Koumboulis, Dimitrios G. Fragkoulis, Nikolaos D. Kouvakas, Aikaterini Feidopiasti

**Affiliations:** 1 Department of Digital Industry Technologies, School of Science, National and Kapodistrian University of Athens, Euripus Campus, 34400 Euboea, Greece; 2 Core Department, National and Kapodistrian University of Athens, Euripus Campus, 34400 Euboea, Greece

**Keywords:** data-driven soft sensor design, data-driven observer design, switching observers, nonlinear processes

## Abstract

The goal of the paper is the design of soft sensors for single input single output (SISO) nonlinear processes. This goal is of essential importance for process monitoring, fault detection and fault isolation. The observer-based technique, being a fruitful direction in soft sensor design, is followed to develop soft sensors for nonlinear processes with known dynamics and unknown physical parameters. A new and general approach, based on the identified I/O linear approximant system descriptions, around prespecified operating points, and a bank of switching linear observers, will be developed. The system property of the I/O reconstructability of the state space linear approximant of a nonlinear model is presented. The design of each observer is based on the I/O measurements and structural characteristics of the nonlinear process. Observer-oriented target areas are introduced, and the respective dense web principle is formulated. The design is completed by the design of a data-driven rule-based system, providing stepwise switching among the observers of the bank. The number of observers of the bank is equal to the number of the linear approximants of the nonlinear process model and is equal to the number of the respective target operating areas. The target operating areas are required to satisfy the dense web principle. The information provided by the soft sensor is the estimation of the non-measured variables of the process. The information used by the soft sensor is the identified I/O approximants of the process as well as the real time values of the measurement variables. The efficiency of the design scheme is illustrated through symbolic and numerical simulation results for a chemostat. The nonlinear model of the chemostat is initially approximated by a set of ten linear approximants. After, the I/O approximants are identified, the respective observers are designed and the target operating areas are determined, where several cases of the satisfaction of the dense web principle are investigated. The soft sensor is composed in terms of the designed observers. Simulation results illustrate the satisfactory performance of the designed soft sensor.

## 1. Introduction

In modern industrial processes, command-and-control systems are purely computerized in a high level aiming towards Industry 4.0 directions and focusing on the satisfaction of multi target goals, simultaneously. Satisfactory performance of the process, high quality products, minimization of the energy consumption and fulfilment of environmental standards are some of these goals. To accomplish these goals, the computerized systems are based on intelligent control and command algorithms based on real time information of several variables of the process. Sensors are devices providing real time measurements of physical variables to the command-and-control system.

In modern industry, soft-sensors are usually installed to indirectly measure difficult-to-measure variables with physical sensors [1]. In most industrial processes, due to inherent technical characteristics or cost limitations, the participating variables are not all measurable in real time. Indicatively, it is mentioned that the concentration of chemical elements is usually measured through laboratory tests, providing sampled and delayed values of the quantity under measurement. Since in most processes the variable sensors are not all available, control design is greatly benefited by the development of soft sensors. Alternatively, control design schemes using subsets of the system variables are required to be developed, indicatively see [2,3,4].

A soft sensor of a non-measured, or not accurately measured, physical variable is an algorithm implemented to the computer system and provides real time estimations of the variable, using real time data of other variables and offline measurement data of the same and/or other variables, as well as appropriate physical or data-driven models. This way, installation of soft sensors in modern industry tackles problems of installation of physical sensors with high calibration and maintenance costs [5] as well as technical problems of difficult to install physical sensors, large measurement delays, etc. [1]. Since soft sensors are special designed software, they are implemented on appropriate computer devices or more preferably, from the industrial control point of view, on industrial embedded systems [5].

There are two main directions in the development of soft sensors. The first one is based on mathematical models, obtained through physical knowledge of the process. Such first-principle-based models require sufficient knowledge about the underlying principles of physics, chemistry and even biology of the system. In some cases, the construction of such mathematical models is overcomplicated requiring significant effort to develop. In all cases, the direct use of these models requires the exact knowledge of all parameters of the developed model. The latter requirement is rarely satisfied in industrial practice. The second direction is the data-driven techniques based on real-time measurements and empirical or experimental data of the process. Although data-driven techniques describe real conditions of the process, they require intensive processing of data ([5,6]) as well as the construction of data-driven models. According to [6], the most popular data-driven models used in soft sensors applications are linear models [7], support vector regressions [8], fuzzy and neuro-fuzzy systems [9]. Several data-driven models are derived through identification (online or offline) using observers (see [10,11]), or machine learning techniques where the basic assumption is that the outputs are uniquely determined by the nonlinear projection of the inputs and/or the dynamics of the systems that approximate some natural behavior (see [12,13]). Many models are integrated using optimization procedures, such as random forest and generic algorithm [14] as well as Kalman filters and Extended Kalman filters covering certain stochastic properties of measurements and noisy environments (see [15,16]).

In the development of soft sensors for process variables, there are two issues of great importance. The first is the distance between the output of the soft sensors in real time, namely the estimation error, and the second is the lack of models of the process. Both issues are treated in the present paper by developing a stepwise switching observer design approach.

Switching observer design as well as observer design for switching systems have attracted considerable interest from various points of view and using different approaches (indicatively, see [17,18,19,20,21] and the references therein). In [17] an observer design for linear superdetectable switched systems has been proposed. In [18,19], observer design techniques for discrete-time linear systems have been proposed. In [18], a hybrid asymptotic observer has been proposed. In [19], three Luenberger-type observers using an LMI technique, have been designed. In [20,21], the stepwise switching observer design has been investigated for the case of two nonlinear process applications. Safe stepwise switching was introduced and established in [22] for the pure control problem of SISO systems. It is mentioned that in the pure control problem, in [22], observers are not participating and neither are used. The results in [22] have been extended in [23] to cover the multi-input multi-output (MIMO) case. In [24], the quite interesting case of two inputs and two outputs (TITO) systems with decoupled linear approximants is studied through a quadrotor application. The stepwise safe switching design for the pure control problem has been studied in several applications (indicatively see [22,23,24,25]). The problem of stepwise safe switching observer design has not yet been formulated in its general form and its solution has not yet been derived.

In the present paper, the problem of designing a stepwise safe switching observer is studied for the case of SISO nonlinear processes where the structure of the nonlinear model of the process is known while the parameters of the model are not considered to be known. Additionally, the operating trajectory of the process is considered to be known. Firstly, for any operating point of the process, a full order linear observer, depending upon a nominal operating point of the process, is considered, using the respective linear approximant of the process. A set of system properties and definitions required for the present analysis are introduced. A bank of observers is designed. Each observer corresponds to a different operating point. The observer design is based on the respective linear approximants of the process model at different operating points. An important aspect of the present results is that the design of the observer is based entirely upon the I/O linear approximant of the process model, being derived through standard identification approaches (indicatively see [26,27]) using I/O data. This way, a look up table of I/O relations, derived through identification, is mapped to a bank of observers. From this point of view, the observers of the bank are data driven observers, in the online and the offline sense of the term, the observers are fed with real time measurements. In the offline sense of the term, the observers are designed using identification data. This type of data-driven observers is the first contribution of the paper.

A supervisor, orchestrating the switching among the observers of the bank, is designed. The goal of the supervisor is to compose an estimation of the state variables that approximates as close as possible the state variables of the nonlinear process model. This is the second contribution of the present paper. The proximity of the estimated variables to the state variables of the nonlinear process is defined and proved through the introduction of appropriate cost criteria, depending upon the identified coefficients of the I/O linear approximants of the process and the operating points of the process.

A new set of target operating areas, oriented to observer design is introduced and the respective dense web principle for observer design is also introduced. This is the third contribution of the paper.

The performance of the proposed design scheme is demonstrated in the case of a chemostat, being a quite applicable system (indicatively see [28,29,30]). The linear approximants of the chemostat model and the respective linear full order observer are analytically determined. The identified I/O linear approximants of the process are derived. The target operating areas of the chemostat model are also derived. Finally, using a series of computational experiments, the satisfactory performance of the proposed design scheme is demonstrated. It is important to mention that the present estimated variables are quite near to the respective estimated variables derived in [28], where a nonlinear observer has been designed, considering that the parameters of the chemostat nonlinear model are accurately known. 

The proposed, here, stepwise safe switching observer scheme is easily implementable to the control-and-command computer system providing the real time data of sensors measuring only some of the variables of the process. The algorithm, realizing the present design scheme, is simple and elegant, in the sense that includes a bank of linear observer orchestrated by a simple supervisor rule. Overall, the proposed algorithm is an adequate soft sensor for industrial processes. It is important to mention that the present results are offered for implementation to low-level computer platforms, such as microcontrollers (μCs), Programmable Automation Controllers (PACs) and other microprocessor embedded systems. 

To facilitate reader’s familiarization with the concepts and procedures introduced, here, the special case of a chemostat model is first investigated. Particularly, in Section 2 the structure of the nonlinear model of the process is presented. In Section 3, the accuracy of the linear approximant of the chemostat is investigated. In Section 4, the full order observer design problem for the linear approximant of the chemostat is investigated. In Section 5, the general framework for linear observer design through parameter identification of SISO I/O linear approximants is developed. In Section 6, the general framework for switching observer design through parameter identification of SISO I/O linear approximants is developed and illustrated through extensive computational experiments.

## 2. The Nonlinear Model of a Chemostat

According to [28,29,30], the general nonlinear model of a chemostat is expressed by the following two nonlinear differential equations:

(1) $$\frac{ds(t)}{dt}=q(t)(s_{in}-s(t))-\frac{1}{\delta }f\left(s(t)\right)x(t),\;\frac{dx(t)}{dt}=f\left(s(t)\right)x(t)-q(t)x(t)$$

where s(t) is the substrate’s concentration, x(t) is the microorganisms’ concertation and q(t) is the dilution rate in the chemostat, being the actuatable input variable of the system. The parameter $s_{in}$ is the concentration of the input substrate. According to [28,29,30], this parameter is constant. The parameter δ is the yield constant. This parameter is considered to be the known (see [28,29,30]). The function $f:{\mathbb{R}}^{+}\to {\mathbb{R}}^{+}$ is the uptake function, being a monotonically increasing, continuously differential and homogeneous function, i.e., f(0)=0. In accordance with [28], it is considered here that the uptake function follows a Monod growth rate form, i.e., it holds that $f\left(s(t)\right)=\mu_{m}s(t)/\left(s(t)+K\right)$, where $\mu_{m}$ is the maximum growth rate of the microorganisms and K is the half saturation constant. Using this consideration, the nonlinear state space model of the chemostat is of the form:

(2) $$\frac{dx_{1}(t)}{dt}=u(t)(s_{in}-x_{1}(t))-\frac{\mu_{m}x_{1}(t)x_{2}(t)}{\delta \left(x_{1}(t)+K\right)},\;\frac{dx_{2}(t)}{dt}=\frac{\mu_{m}x_{1}(t)x_{2}(t)}{\left(x_{1}(t)+K\right)}-u(t)x_{2}(t),$$

where $x_{1}(t)=s(t)$ and $x_{2}(t)=x(t)$. The state vector is $\tilde{x}(t)={\left[\begin{array}{cc} x_{1}(t) & x_{2}(t) \end{array}\right]}^{T}$. The measurement output variable is $y(t)=x_{1}(t)=s(t)$. The control input variable is u(t)=q(t). The initial condition of the system is $\tilde{x}(0-)={\tilde{x}}_{0}$.

Let $Y,\tilde{X}$ and U be the operating values of the measurement output variable, the state vector, and the control input variable, respectively. The operating values of the state vector and the performance variable are expressed as functions of the operating value of the input, as follows:

(3a) $$\tilde{X}=\left[\begin{array}{c} X_{1}\\ X_{2} \end{array}\right]=\left[\begin{array}{c} KU/(\mu_{m}-U)\\ \delta \left(s_{in}-\frac{KU}{\mu_{m}-U}\right) \end{array}\right],$$

(3b) $$Y=KU/(\mu_{m}-U).$$

Relation (3b) is the operating trajectory of the chemostat model, relating the operating values of the control input to the operating values of the measurement output.

## 3. The Linear Approximants of the Chemostat

The linear approximant of the chemostat model, around the operating values of the input the output and the state variables, is computed to be in the following general state space form, depending entirely upon the operating value of the input,

(4) $$\begin{array}{cc} ℵ: & \begin{array}{l} \Delta {\dot{x}}_{L}(t)=A\Delta x_{L}(t)+b\Delta u(t),\;\Delta y_{L}(t)=c\Delta x_{L}(t),\\ \Delta x_{L}(0-)=\Delta x_{L,0}={\tilde{x}}_{0}-\tilde{X} \end{array} \end{array}$$

where the system matrices are

(5a) $$A=\left[\begin{array}{cc} -\frac{KU^{2}+s_{in}{\left(\mu_{m}-U\right)}^{2}}{K\mu_{m}} & -\frac{U}{\delta }\\ \frac{\delta \left(\mu_{m}-U\right)\left[s_{in}\left(\mu_{m}-U\right)-KU\right]}{K\mu_{m}} & 0 \end{array}\right],\;b=\left[\begin{array}{c} s_{in}-\frac{KU}{\mu_{m}-U}\\ \frac{KU\delta }{\mu_{m}-U}-s_{in}\delta \end{array}\right],\;c=\left[\begin{array}{cc} 1 & 0 \end{array}\right],$$

(5b) $$\Delta x_{L}(t)={\left[\begin{array}{cc} \Delta x_{L,1}(t) & \Delta x_{L,2}(t) \end{array}\right]}^{T},$$

and where $\Delta y_{L}(t)$, $\Delta x_{1}(t)$ and $\Delta x_{2}(t)$ are the approximants of the deviations Δy(t)=y(t)−Y, $\Delta x_{1}(t)=x_{1}(t)-X_{1}$ and $\Delta x_{2}(t)=x_{2}(t)-X_{2}$, respectively. The input of the linear approximant is the deviation Δu(t)=u(t)−U.

The general form of the I/O description of the linear approximant (4) of the model of the chemostat, namely the I/O linear approximant of the process, is

(6) $$S:\;\;\;\Delta y_{L}^{\left(1\right)}(t)+h_{D}\Delta y_{L}(t)=h_{N}\Delta u(t)$$

In the following relations, the coefficients of (6) are expressed in terms of the system parameters:

(7) $$h_{D}=\frac{\left[s_{in}\left(\mu_{m}-U\right)-KU\right]\left(\mu_{m}-U\right)}{K\mu_{m}},\;h_{N}=\frac{\mu_{m}s_{in}-(K+s_{in})U}{\mu_{m}-U}.$$

If the system parameters $s_{in}$, $\mu_{m}$ and K are not known, while the operating values Y and U as well as the coefficients of the I/O model (6), $h_{D}$ and $h_{N}$ are known, then the system parameters are determined by the analytic expressions

(8) $$\mu_{m}=\frac{h_{N}U^{2}}{h_{N}U-h_{D}Y},\;s_{in}=h_{N}+Y,\;K=\frac{h_{D}Y^{2}}{h_{N}U-h_{D}Y}.$$

As already mentioned, the yield constant δ is considered to be known. Hence, substituting (8) to (5a), the system matrices A and b are expressed as functions of the coefficients of the I/O model and the operating value of the input as follows:

(9) $$A(h_{D})=\left[\begin{array}{cc} -h_{D}-U & -U/\delta \\ h_{D}\delta & 0 \end{array}\right],\;b(h_{N})=\left[\begin{array}{c} h_{N}\\ -\delta h_{N} \end{array}\right].$$

Additionally, the operating state vector in (3a) is expressed by the relation

(10) $$\tilde{X}=\left[\begin{array}{c} X_{1}\\ X_{2} \end{array}\right]=\left[\begin{array}{c} Y\\ h_{N}\delta \end{array}\right].$$

It is important to mention that according to [28,29,30,31], the dilution rate has an upper bound, denoted by $U_{\max}$ and a lower bound denoted by $U_{\min}$. Both bounds depend upon technical characteristics of the process. According to [32], the dilution rate’s upper bound in the normal operation of the chemostat is $U_{\max}\approx \mu_{m}$. On the contrary, if U is very small then the culture will be washed out (see [32,33]). According to [32], the dilution rate varies between $U_{\min}=0.005$ and $U_{\max}=1$. According to [34], the operating variable of the input variable is constrained to satisfy the inequalities:

(11a) $$U>0,\;U<\mu_{m},U<\frac{s_{in}}{K+s_{in}}\mu_{m}.$$

Considering that K, $s_{in}$ and $\mu_{m}$ are positive parameters, the previous inequalities reduce to

(11b) $$0<U<\frac{s_{in}}{K+s_{in}}\mu_{m}.$$

In concluding, for the satisfactory performance of the chemostat, it holds that

(11c) $$U\in (U_{\min},U_{\max})\;;\;U_{\min}=0,\;U_{\max}=\frac{s_{in}}{K+s_{in}}\mu_{m}.$$

It can readily be verified that under the upper and lower constraints in (11c), the nominal values of the state variables satisfy the following inequalities:

(11d) $$X_{1}\in (X_{1,\min},X_{1,\max})\;;\;X_{1,\min}=0,\;X_{1,\max}=s_{in},$$

(11e) $$X_{2}\in (X_{2,\min},X_{2,\max})\;;\;X_{2,\min}=0,\;X_{2,\max}=s_{in}\delta .$$

The characteristic polynomial of the linear approximant can be rewritten in the form $p\left(s\right)=\left(s-r_{1}\right)\left(s-r_{2}\right)$, where

(12a) $$r_{1}=-U,\;r_{2}=-\frac{\left[s_{in}\left(\mu_{m}-U\right)-KU\right]\left(\mu_{m}-U\right)}{K\mu_{m}}.$$

The above roots of the characteristic polynomial are expressed in terms of the I/O approximant and the operating value of the input variable as follows:

(12b) $$r_{1}=-U,\;r_{2}=-h_{D}$$

Considering that $\mu_{m}$, $s_{in}$ and K are positive as well as the constraints in (11c), it can be observed that $r_{1}$ and $r_{2}$ are always negative. Hence, the linear approximant (4) is asymptotically stable. Thus, local asymptotic stability for the original nonlinear model (2) (see also [35]) is guaranteed.

To quantitatively evaluate the accuracy of the sum of the forced and the free response of the linear approximant of the process, in comparison to the respective response of the nonlinear process, the three criteria are required to be small enough simultaneously. Define

(13a) $$J_{\infty }=\frac{\underset{j=1,2}{\max}\left\{\underset{t\in \left[t_{0},{\left(T_{\max}\right)}_{j}\right]}{\sup}\left|x_{j}(t)-\Delta x_{L,j}(t)-X_{j}\right|\right\}}{\underset{j=1,2}{\max}\left\{\underset{t\in \left[t_{0},{\left(T_{\max}\right)}_{j}\right]}{\sup}\left|\Delta x_{L,j}(t)\right|\right\}}\times 100\%,$$

(13b) $$J_{1}={\left(\frac{\sum_{j=1}^{2}{\left[\underset{t\to +\infty }{\lim}\left|x_{j}(t)-\Delta x_{L,j}(t)-X_{j}\right|\right]}^{2}}{\sum_{j=1}^{2}{\left[\underset{t\to +\infty }{\lim}\left|\Delta x_{L,j}(t)\right|\right]}^{2}}\right)}^{\frac{1}{2}}\times 100\%,$$

(13c) $$J_{2}={\left(\frac{\sum_{j=1}^{2}\int_{t_{0}-}^{{\left(T_{\max}\right)}_{j}}{\left[x_{j}(t)-\Delta x_{L,j}(t)-X_{j}\right]}^{2}dt}{\sum_{j=1}^{2}\int_{t_{0}-}^{{\left(T_{\max}\right)}_{j}}{\left[\Delta x_{L,j}(t)\right]}^{2}dt}\right)}^{\frac{1}{2}}\times 100\%.$$

The parameter ${\left(T_{\max}\right)}_{j}$ is selected to be equal to the time required for $x_{j}(t)$ to settle in an area around 2% of its steady state value.

Given the operating points of the actuatable input and the state variables, the actuatable input for the nonlinear and linear approximant will be selected to be in the form

(14) $$u(t)=U\left(1+p_{u}u_{s}\left(t-t_{0}\right)\right),\;\Delta u(t)=p_{u}Uu_{s}\left(t-t_{0}\right),$$

where $u_{s}(t)$ is the unit step signal, $p_{u}\in \left({\left(p_{u}\right)}_{\min},{\left(p_{u}\right)}_{\max}\right)$, ${\left(p_{u}\right)}_{\min},{\left(p_{u}\right)}_{\max}\in \mathbb{R}$ and

(15) $$-1<{\left(p_{u}\right)}_{\min}<{\left(p_{u}\right)}_{\max}.$$

The norms in (13) will be evaluated for various actuatable input signals and various initial conditions of the nonlinear model and its linear approximant, through a series of computational experiments. The initial values of the nonlinear model and linear approximant will be selected to be

(16) $$x_{j}(t_{0}-)=\left(1+p_{j}\right)X_{j}\;,\;\Delta x_{L,j}(t_{0}-)=p_{j}X_{j};\;j=1,2,$$

where $p_{j}\in \left({\left(p_{j}\right)}_{\min},{\left(p_{j}\right)}_{\max}\right)$, ${\left(p_{j}\right)}_{\min},{\left(p_{j}\right)}_{\max}\in \mathbb{R}$ and $-1<{\left(p_{j}\right)}_{\min}<{\left(p_{j}\right)}_{\max}$.

Since the nonlinear model (2) is locally asymptotically stable and the linear approximant (4) is asymptotically stable, it is observed that the cost $J_{1}$, defined in (13b), does not depend upon the initial conditions but only upon the steady state value of the actuatable input. Using (14) and after appropriate algebraic manipulations, the following relation is derived

(17) $$J_{1}=\left|\frac{p_{u}U}{\left(1+p_{u}\right)U-\mu_{m}}\right|\times 100\%.$$

Let $\varepsilon_{1}\in {\mathbb{R}}^{+}$ be an upper bound for $J_{1}$, set by the designer, i.e., the following inequality is required to be satisfied

(18) $$J_{1}<\varepsilon_{1}.$$

For a given $\varepsilon_{1}$, using the inequalities in (11b), the following scenarios for the selection of $p_{u}$ are derived:

- If $\varepsilon_{1}<100\%$, then $\frac{\left(\mu_{m}-U\right)\varepsilon_{1}}{\left(\varepsilon_{1}-1\right)U}<p_{u}<\frac{\left(\mu_{m}-U\right)\varepsilon_{1}}{\left(\varepsilon_{1}+1\right)U}$.
- If $\varepsilon_{1}=100\%$, then $p_{u}<\frac{\mu_{m}-U}{2U}$.
- If $\varepsilon_{1}>100\%$, then $\left(p_{u}<\frac{\left(\mu_{m}-U\right)\varepsilon_{1}}{\left(\varepsilon_{1}+1\right)U}\right)\vee \left(p_{u}>\frac{\left(\mu_{m}-U\right)\varepsilon_{1}}{\left(\varepsilon -1\right)U}\right)$.

In a similar manner, the linear approximant (4) is an accurate representation of the nonlinear model (2), regarding the metrics defined in (3a) and (13c), if

(19) $$J_{2}<\varepsilon_{2},$$

(20) $$J_{\infty }<\varepsilon_{\infty },$$

where $\varepsilon_{2}\in {\mathbb{R}}^{+}$ and $\varepsilon_{\infty }\in {\mathbb{R}}^{+}$ are the desirable upper bounds for $J_{2}$ and $J_{\infty }$. 

To execute the computational experiments, examining the accuracy of the linear approximant (4) as compared to the nonlinear model (2), the following chemostat data, presented in [28,36], are used δ=1/6.6 [kg biomass/kg COD], $\mu_{m}=1.2\;\;[{\mathrm{day}}^{-1}]$, $K=4{.95\;[\mathrm{kg}\;\mathrm{COD}/m}^{3}]$ and $s_{in}=9\;\;[{\mathrm{kg}/m}^{3}]$. Using the above data and (11c), we obtain $U_{\min}=0[{\mathrm{day}}^{-1}]$, $U_{\max}=0.7742[{\mathrm{day}}^{-1}]$. 

In what follows, a series of computational experiments will be conducted, for different operating conditions and various values of $p_{u}$, $p_{1}$ and $p_{2}$. In particular, the ten scenarios of nominal conditions presented in Table S1 of Supplementary Material (Supplementary Material) of the paper, covering satisfactorily the range of the nominal values of the input variable, will be studied. In these scenarios, the operating values of the input and the state variables are presented. The ten scenarios have been computed using (3a) and the inequality constraints in (11c)–(11e).

**Figure 1:**
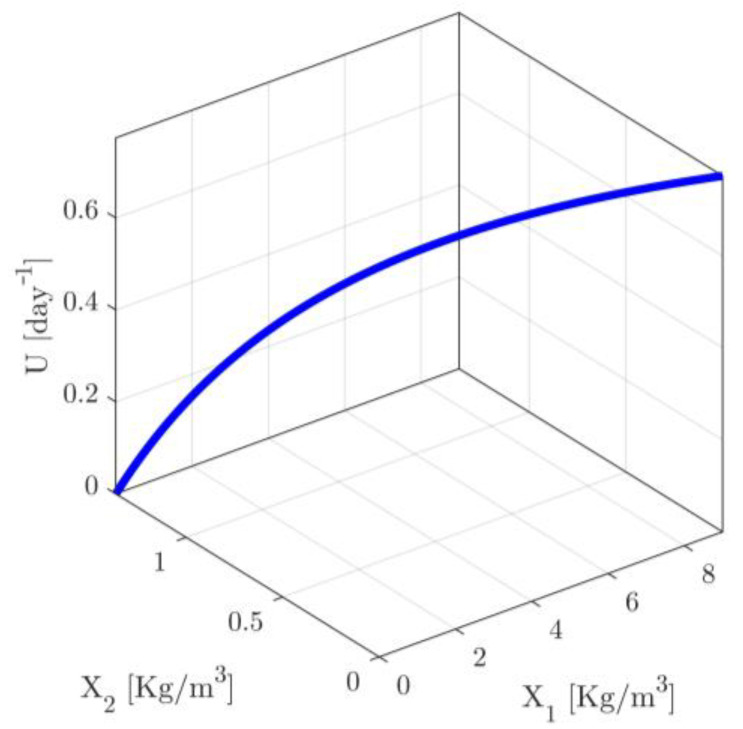
Operating Trajectory of the Chemostat.

For all scenarios presented in Table S1 of Supplementary Material, the actuatable input and the initial conditions of the state variables are considered to vary in intervals with the following starting and ending points ${\left(p_{u}\right)}_{\min}=-1$, ${\left(p_{u}\right)}_{\max}=\frac{U_{\max}}{U}-1$, ${\left(p_{j}\right)}_{\min}=-1$ and ${\left(p_{j}\right)}_{\max}=\frac{X_{j,\max}}{X_{j}}-1$ (j=1,2). Note that the minimum and maximum values of $p_{u}$ correspond to actuatable input signals in the limits given in (11c), while the minimum and maximum values of $p_{j}$ satisfy the respective limits in (11d,e). Finally, the accuracy thresholds are chosen to be enough small, i.e., they are chosen to be $\varepsilon_{1}=5\%$, $\varepsilon_{2}=5\%$ and $\varepsilon_{\infty }=5\%$. It is plausible to expect that the areas of accuracy will be extended around the operating trajectory of the process (see Figure 1). Note that, in industrial practice the operating trajectory of a process is usually considered to be known through appropriate small scale sampled experimentation and then data interpolation, in cases where the system parameters are not accurately known to the designer.

**Figure 2:**
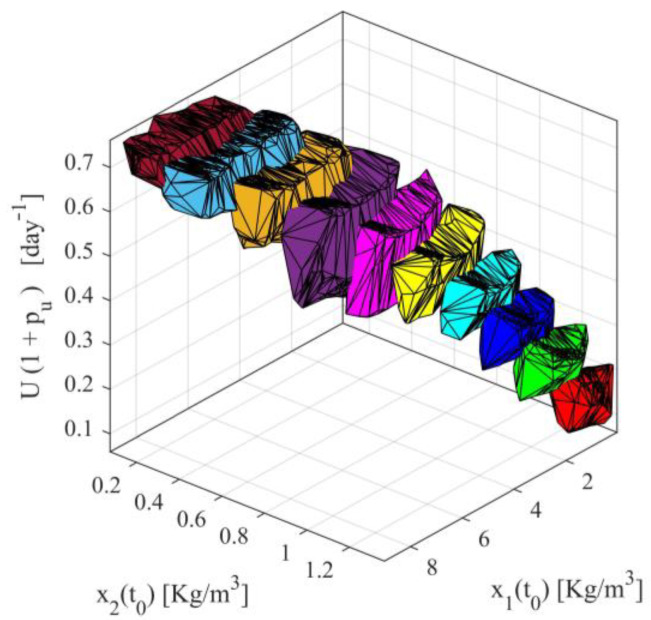
Acceptable input and initial condition sets for all scenarios.

Applying series of computations, the accuracy areas for all ten scenarios, namely the areas satisfying simultaneously the conditions (18)–(20), are presented in Figure 2, as ten three dimensional volumes with different colors. It can readily be observed that for each operating point there exists a wide range of inputs and initial condition satisfying the accuracy conditions (18)–(20). Furthermore, it is observed there is overlapping between adjacent volumes. In the overlapping, the accuracy conditions are simultaneously satisfied for the adjacent operating points.

## 4. Observer Design Using the Coefficients of the I/O Linear Approximant of the Chemostat

### 4.1. Observer Design

The observability matrix of the linear approximant ℵ is

(21) $$O=\left[\begin{array}{cc} 1 & 0\\ -\left[KU^{2}+s_{in}{\left(U-\mu_{m}\right)}^{2}\right]/\left(K\mu_{m}\right) & -U/\delta \end{array}\right]=\left[\begin{array}{cc} 1 & 0\\ -h_{D}-U & -U/\delta \end{array}\right].$$

From (22), it can readily be observed that ℵ is observable if and only if U≠0, imposed by (13c). Hence, the observability of the linear approximant is independent from the coefficients of the I/O linear approximant and the operating value of the input variable. 

The full order observer of ℵ is the following linear system

(22) $$ℑ:\Delta {\dot{\hat{x}}}_{L}(t)=F\Delta {\hat{x}}_{L}(t)+g\Delta y_{L}(t)+m\Delta u(t),\;\;\;\Delta {\hat{x}}_{L}(0-)=\Delta {\hat{x}}_{L,0},$$

where $\Delta {\hat{x}}_{L}(t)\in {\mathbb{R}}^{2\times 1}$ is the estimation of the state vector of ℵ, namely the estimation of $\Delta x_{L}(t)$. The estimation error of the full order observer (22) is defined to be

(23) $$e_{L}(t)=\Delta x_{L}(t)-\Delta {\hat{x}}_{L}(t).$$

The estimation of the original state variable vector $\tilde{x}(t)$ is proposed to be

(24) $$\hat{x}(t)=\Delta {\hat{x}}_{L}(t)+\tilde{X}=\Delta {\hat{x}}_{L}(t)+\left[\begin{array}{cc} Y & h_{N}\delta \end{array}\right].$$

The estimation error of the state vector of $\tilde{x}(t)$ is

(25) $$e(t)=\tilde{x}(t)-\hat{x}(t)=\Delta \tilde{x}(t)-\Delta {\hat{x}}_{L}(t);\;\Delta \tilde{x}(t)=\tilde{x}(t)-\tilde{X}.$$

The general forms of the observer gain matrices, in terms of the physical parameters of the system, are

$$g={\left[\begin{array}{cc} g_{1} & g_{2} \end{array}\right]}^{T},\;m=b=\left[\begin{array}{c} s_{in}-\frac{KU}{\mu_{m}-U}\\ \frac{KU\delta }{\mu_{m}-U}-s_{in}\delta \end{array}\right],\;F=F(g_{1},g_{2})=A-gc=\left[\begin{array}{cc} -\frac{KU^{2}+s_{in}{\left(\mu_{m}-U\right)}^{2}}{K\mu_{m}}-g_{1} & -\frac{U}{\delta }\\ \frac{\delta \left(\mu_{m}-U\right)\left[s_{in}\left(\mu_{m}-U\right)-KU\right]}{K\mu_{m}}-g_{2} & 0 \end{array}\right]$$

or alternatively in terms of the coefficients of the I/O linear approximant

(26) $$F=F(h_{D},g_{1},g_{2})=\left[\begin{array}{cc} -h_{D}-U-g_{1} & -U/\delta \\ h_{D}\delta -g_{2} & 0 \end{array}\right],\;m(h_{n})=\left[\begin{array}{c} h_{N}\\ -\delta h_{N} \end{array}\right].$$

The estimation error of the linear approximant is governed by the equation

(27) $${\dot{e}}_{L}(t)=Fe_{L}(t).$$

The characteristic polynomial of F is

(28a) $$\det\left(sI_{2}-F\right)=s^{2}+a_{f,1}s+a_{f,0},$$

where

(28b) $$a_{f,1}=\frac{KU^{2}+s_{in}{\left(\mu_{m}-U\right)}^{2}}{K\mu_{m}}+g_{1}=h_{D}+U+g_{1},\;a_{f,0}=\frac{U\left[s_{in}\left(\mu_{m}-U\right)-KU\right]\left(\mu_{m}-U\right)}{K\mu_{m}}-\frac{U}{\delta }g_{2}=Uh_{d}-\frac{U}{\delta }g_{2}$$

To achieve enough small estimation error, the requirement adopted here is regional stability of F. Consider the a− regional stability, i.e., that the eigenvalues of F must belong to ℂa−=s∈ℂ:Res<−a, where a is a non-negative real number, i.e., $a\in {\mathbb{R}}_{0}^{+}=\{\alpha \in \mathbb{R}:\alpha \geq 0\}$. This property is satisfied if and only if the following inequalities are satisfied

$$g_{1}>-\frac{s_{in}{\left(\mu_{m}-U\right)}^{2}+K\left(U^{2}-2a\mu_{m}\right)}{K\mu_{m}},\;g_{2}<\frac{\delta \left(U-a\right)\left\{s_{in}{\left(\mu_{m}-U\right)}^{2}+K\left[U^{2}-\left(U+a\right)\mu_{m}\right]\right\}}{KU\mu_{m}}-\frac{a\delta }{U}g_{1},$$

or equivalently if and only if

(29a) $$g_{1}>2a-h_{D}-U$$

(29b) $$g_{2}<\frac{\left(h_{D}-a\right)\left(U-a\right)\delta }{U}-\frac{a\delta }{U}g_{1}$$

Additionally, the requirement that the roots of the characteristic polynomial of F are real and distinct, is adopted here. For this additional requirement to be satisfied, it must hold that $a_{f,1}^{2}-4a_{f,0}>0$, or equivalently that

(30) $$g_{2}>-\frac{\delta }{4U}\left[{\left(h_{D}-U\right)}^{2}+2\left(h_{D}+U\right)g_{1}+g_{1}^{2}\right].$$

Assuming that the inequalities in (29a,b) and (30) hold simultaneously, namely the observer characteristic polynomial has real and distinct roots, being a− regional stable, the response of the estimation error dynamics in (27), is of the form

(31) $$e_{L}(t)=\Phi \left(t\right)e_{L,0},$$

where $\Phi \left(t\right)$ is the transition matrix of (25), being is the inverse Laplace transform of the resolvent matrix ${\left(sI_{n}-F\right)}^{-1}$. Clearly $\Phi \left(t\right)$ can be expressed in the form $\Phi \left(t\right)=e^{-\rho_{F,1}t}\Phi_{1}+e^{-\rho_{F,2}t}\Phi_{2}$, where $\;\Phi_{1}$ and $\Phi_{2}$ are two by two real matrices and, $-\rho_{F,1}$ and $-\rho_{F,2}$ are the eigenvalues of the observer matrix F, where $\min\left\{\rho_{F,1},\rho_{F,2}\right\}>a$. 

The convergence rate of the error dynamics of the observer depends upon the eigenvalues of F. The following metric is proposed to evaluate the rate of convergence, being an upper bound of the transition matrix at the critical time instant t=1,

(32) $$J_{e,A}\left(U\right)=\exp\left(-a\right)\left({\left\|\Phi_{1}\right\|}_{\alpha }+{\left\|\Phi_{2}\right\|}_{\alpha }\right),$$

where ${\left\|\cdot \right\|}_{\alpha }$ denotes the α norm of the argument vector or matrix and α∈{1,2,...,∞}. For observer design purposes, it would be desirable for the metric in (32) to be appropriately bounded, i.e.,

(33) $$J_{e,A}\left(U\right)\leq \zeta_{O,A},$$

where $\zeta_{O,A}\in {\mathbb{R}}^{+}$ is an enough small threshold.

To investigate the steady state behavior of the observer, in comparison to the respective steady state behavior of original nonlinear chemostat model, the following parametric expressions are derived:

(34a) $$\underset{t\to \infty }{\lim}\left|x_{1}(t)-{\hat{x}}_{1}(t)\right|=\left|\frac{h_{N}u_{w}^{2}\left(h_{D}Y-h_{N}U\right)\delta }{\left[h_{N}Uu_{w}-h_{D}\left(U+u_{w}\right)Y\right]\left(h_{D}\delta -g_{2}\right)}\right|,$$

(34b) $$\underset{t\to \infty }{\lim}\left|x_{2}(t)-{\hat{x}}_{2}(t)\right|=\left|\frac{h_{N}u_{w}^{2}\left(h_{N}U-h_{D}Y\right)\delta \left[\delta \left(U+g_{1}\right)+g_{2}\right]}{U\left[h_{N}Uu_{w}-h_{D}\left(U+u_{w}\right)Y\right]\left(h_{D}\delta -g_{2}\right)}\right|.$$

where the steady state value of the input variable is considered to be of the form $\underset{t\to \infty }{\lim}u(t)=U+u_{w}$, where $u_{w}$ is the deviation from the nominal operating value of the input. It is important to mention that for the derivation of (3a) and (34b), the inequalities in (29a,b) have been used together with the stability of the chemostat model. It is noted that for this derivation, the condition (30) has not been used. From (34a,b), it is observed that the initial conditions of the chemostat model and the observer do not affect the steady state values of either system. Finally, the following dependence relation is derived:

$$\underset{t\to \infty }{\lim}\left|x_{1}(t)-{\hat{x}}_{1}(t)\right|=\left|\frac{U}{\delta \left(U+g_{1}\right)+g_{2}}\right|\underset{t\to \infty }{\lim}\left|x_{2}(t)-{\hat{x}}_{2}(t)\right|.$$

To evaluate the differences between steady state behavior of the observer and steady state behavior of original nonlinear chemostat model, the following steady state estimation error metric is proposed:

(35) $$J_{e,O}(h,U,u_{w})=\frac{\sum_{j=1}^{2}{\left[\underset{t\to +\infty }{\lim}\left|x_{j}(t)-\Delta {\hat{x}}_{L,j}(t)-X_{j}\right|\right]}^{2}}{\sum_{j=1}^{2}{\left[\underset{t\to +\infty }{\lim}\left|x_{j}(t)-X_{j}\right|\right]}^{2}}.$$

It can be verified that relation (35) can be rewritten as follows:

(36a) $$J_{e,O}\left(h_{D},h_{N},U,u_{w}\right)=u_{w}^{2}{\tilde{J}}_{e,O}\left(h_{D},h_{N},U\right),$$

where

(36b) $${\tilde{J}}_{e,O}\left(h_{D},h_{N},U\right)={\left(\frac{\delta \left(h_{N}U-h_{D}Y\right)}{U^{2}Y\left(g_{2}-h_{D}\delta \right)}\right)}^{2}\left(U^{2}+\frac{\left(\delta g_{1}+g_{2}\right)\left[\left(U\delta +g_{1}\right)\delta +g_{2}\right]}{1+\delta^{2}}\right)$$

For observer design purposes, it is desirable for the metric in (36a) to be appropriately bounded, i.e., $J_{e,O}\left(h_{D},h_{N},U,u_{w}\right)\leq \zeta_{e,O}$, where $\zeta_{e,O}\in {\mathbb{R}}^{+}$ is an enough small threshold.

### 4.2. The General Solution of the Measurement Output Vector in the Observer Dynamics

Consider the observer characteristic polynomial (26). Τhe general solution of (29a) is of the form

(37) $$g_{1}=2a-h_{D}-U+\gamma_{1}.$$

where $\gamma_{1}\in {\mathbb{R}}^{+}$. Substitution of the general solution (37) to the inequality (29b) yields

(38) $$g_{2}<\left[h_{D}-\frac{a}{U}\left(a+\gamma_{1}\right)\right]\delta .$$

The general solution of the inequality (38) is of the form

(39) $$g_{2}=\delta \left[h_{D}-\frac{a}{U}\left(a+\gamma_{1}\right)-\gamma_{2}\right],$$

where $\gamma_{2}\in {\mathbb{R}}^{+}$. Clearly, (37) and (39) are the general solution of a− regional stability. Substituting (37) and (39) to F and (28b) we obtain

(40) $$F=F(\gamma_{1},\gamma_{2})=\left[\begin{array}{cc} -2a-\gamma_{1} & -\frac{U}{\delta }\\ \frac{\delta a}{U}\left(a+\gamma_{1}\right)+\delta \gamma_{2} & 0 \end{array}\right]$$

and that the coefficients of the characteristic polynomial in (28a,b) become

(41) $$a_{f,1}=2a+\gamma_{1},\;a_{f,0}=a\left(a+\gamma_{1}\right)+U\gamma_{2}$$

Clearly, $a_{f,1}$ and $a_{f,0}$ are positive reals. Hence, the roots of the characteristic polynomial have negative real parts. It is important to mention that relations (37) and (39) constitute the I/O dependent general solution for the measurement output gain vector, while the resulting matrix F is independent of the transmission pole $h_{D}$.

The domain of the free parameters $\gamma_{1}$ and $\gamma_{2}$, for the eigenvalues of F to belong to ℂa−=s∈ℂ:Res<−a and to be real and distinct, is the following set of inequalities

(42) $$\left(\gamma_{1}>0\right)\wedge \left(0<\gamma_{2}<\frac{\gamma_{1}^{2}}{4U}\right).$$

Assuming that (42) holds true, then the steady state estimation errors become

(43a) $$\underset{t\to \infty }{\lim}\left|x_{1}(t)-{\hat{x}}_{1}(t)\right|=\left|\frac{h_{N}Uu_{w}^{2}\left(h_{N}U-h_{D}Y\right)}{\left[h_{N}Uu_{w}+h_{D}\left(U+u_{w}\right)Y\right]\left[a\left(a+\gamma_{1}\right)+U\gamma_{2}\right]}\right|,$$

(43b) $$\underset{t\to \infty }{\lim}\left|x_{2}(t)-{\hat{x}}_{2}(t)\right|=\left|\frac{h_{N}u_{w}^{2}\delta \left(h_{N}U-h_{D}Y\right)\left[U\left(2a+\gamma_{1}-\gamma_{2}\right)-a\left(a+\gamma_{1}\right)\right]}{U\left[h_{N}Uu_{w}-h_{D}\left(U+u_{w}\right)Y\right]\left[\alpha \left(\alpha +\gamma_{1}\right)+U\gamma_{2}\right]}\right|,$$

and relation (36b) becomes

(44) $${\tilde{J}}_{e,O}\left(h_{D},h_{N},,U,u_{w}\right)={\left(\frac{h_{N}U-h_{D}Y}{U^{2}Y\left[a\left(a+\gamma_{1}\right)+U\gamma_{2}\right]}\right)}^{2}\left(\frac{U^{4}+{\left[a\left(a+\gamma_{1}\right)+U\left(\gamma_{2}-2a-\gamma_{1}\right)\right]}^{2}\delta^{2}}{1+\delta^{2}}\right)$$

Additionally, the eigenvalues of F are expressed in the following form

(45) $$\rho_{F,1}=\frac{1}{2}\left(2a+\gamma_{1}+\sqrt{\gamma_{1}^{2}-4U\gamma_{2}}\right),\;\rho_{F,2}=\frac{1}{2}\left(2a+\gamma_{1}-\sqrt{\gamma_{1}^{2}-4U\gamma_{2}}\right)$$

Finally, the two constant matrices of the transition matrix take on the form

(46a) $$\Phi_{1}=\left[\begin{array}{cc} \frac{2a+\gamma_{1}+\sqrt{\gamma_{1}^{2}-4U\gamma_{2}}}{2\sqrt{\gamma_{1}^{2}-4U\gamma_{2}}} & \frac{U}{\sqrt{\gamma_{1}^{2}-4U\gamma_{2}}\delta }\\ -\frac{\left[a\left(a+\gamma_{1}\right)+U\gamma_{2}\right]\delta }{U\sqrt{\gamma_{1}^{2}-4U\gamma_{2}}} & \frac{-2a-\gamma_{1}+\sqrt{\gamma_{1}^{2}-4U\gamma_{2}}}{2\sqrt{\gamma_{1}^{2}-4U\gamma_{2}}} \end{array}\right],$$

(46b) $$\Phi_{2}=\left[\begin{array}{cc} \frac{-2a-\gamma_{1}+\sqrt{\gamma_{1}^{2}-4U\gamma_{2}}}{2\sqrt{\gamma_{1}^{2}-4U\gamma_{2}}} & -\frac{U}{\sqrt{\gamma_{1}^{2}-4U\gamma_{2}}\delta }\\ \frac{\left[a\left(a+\gamma_{1}\right)+U\gamma_{2}\right]\delta }{U\sqrt{\gamma_{1}^{2}-4U\gamma_{2}}} & \frac{2a+\gamma_{1}+\sqrt{\gamma_{1}^{2}-4U\gamma_{2}}}{2\sqrt{\gamma_{1}^{2}-4U\gamma_{2}}} \end{array}\right],$$

To quantify the convergence rate of the error dynamics, the criterion in (32) will be used, for α=2. So, the 2-norms of $\Phi_{1}$ and $\Phi_{2}$ are evaluated through the relations

(47) $${\left\|\Phi_{k}\right\|}_{2}=\sigma_{1}\left(\Phi_{k}\right);\;k=1,2.$$

where $\sigma_{1}\left(•\right)$ denotes the largest singular value of the argument matrix, to be

(48) $$\begin{array}{l} {\left\|\Phi_{k}\right\|}_{2}=\left\{\left[U^{4}+\alpha^{2}\delta^{4}{\left(\alpha +\gamma_{1}\right)}^{2}-2U^{3}\delta^{2}\gamma_{2}+2U\alpha \delta^{4}\left(\alpha +\gamma_{1}\right)\gamma_{2}+\right.\right.\\ {\left.\left.+U^{2}\delta^{2}\left(2\alpha^{2}+2\alpha \gamma_{1}+\gamma_{1}^{2}+\delta^{2}\gamma_{2}^{2}\right)\right]/\left[U^{2}\delta^{2}\left(\gamma_{1}^{2}-4U\gamma_{2}\right)\right]\right\}}^{1/2} \end{array};\;k=1,2.$$

From relations (32) and (48), it can readily be verified that

(49) $$\begin{array}{l} J_{e,A}\left(U\right)=2\exp\left(-a\right)\left\{\left[U^{4}+\alpha^{2}\delta^{4}{\left(\alpha +\gamma_{1}\right)}^{2}-2U^{3}\delta^{2}\gamma_{2}+2U\alpha \delta^{4}\left(\alpha +\gamma_{1}\right)\gamma_{2}+\right.\right.\\ {\left.\left.+U^{2}\delta^{2}\left(2\alpha^{2}+2\alpha \gamma_{1}+\gamma_{1}^{2}+\delta^{2}\gamma_{2}^{2}\right)\right]/\left[U^{2}\delta^{2}\left(\gamma_{1}^{2}-4U\gamma_{2}\right)\right]\right\}}^{1/2} \end{array}$$

Using (42), an alternative form of the above expressions can be derived by expressing $\gamma_{1}$ and $\gamma_{2}$ with respect to $\rho_{F,1}$ and $\rho_{F,2}\in {\mathbb{R}}^{+}$, being the eigenvalues of F, can be derived

(50) $$\gamma_{1}=\rho_{F,1}+\rho_{F,2}-2a,\;\gamma_{2}=\left(a-\rho_{F,1}\right)\left(a-\rho_{F,2}\right)U^{-1}.$$

The alternative expressions are

(51) $$F=\left[\begin{array}{cc} -\rho_{F,1}-\rho_{F,2} & -\frac{U}{\delta }\\ \frac{\delta \rho_{F,1}\rho_{F,2}}{U} & 0 \end{array}\right],$$

(52) $${\tilde{J}}_{e,O}\left(h_{D},h_{N},,U,u_{w}\right)={\left(\frac{h_{N}U-h_{D}Y}{U^{2}Y\rho_{F,1}\rho_{F,2}}\right)}^{2}\left(\frac{U^{4}+\delta^{2}\rho_{F,1}^{2}\rho_{F,2}^{2}-2U\delta^{2}\rho_{F,1}\rho_{F,2}\left(\rho_{F,1}+\rho_{F,2}\right)+U^{2}\delta^{2}{\left(\rho_{F,1}+\rho_{F,2}\right)}^{2}}{1+\delta^{2}}\right)$$

(53) $$J_{A}\left(U\right)=2e^{-a}\sqrt{\frac{\left(U^{2}+\delta^{2}\rho_{F,1}^{2}\right)\left(U^{2}+\delta^{2}\rho_{F,2}^{2}\right)}{U^{2}\delta^{2}{\left(\rho_{F,1}-\rho_{F,2}\right)}^{2}}}.$$

To illustrate the performance of the above proposed observer design of the chemostat and demonstrate the derivation of the observer parameters $\rho_{F,1}$ and $\rho_{F,2}$, extensive series of computations are executed. In particular, the goal is to compute $\rho_{F,1}$ and $\rho_{F,2}$ such that (52) is minimized, under the constraints (33), while $0\leq a<\rho_{F,1}<\rho_{F,1}$. The stability margin a is selected such that $a>\underset{i=\left\{1,\ldots ,10\right\}}{\max}\left\{U_{i},{\left(h_{D}\right)}_{i}\right\}$, where $U_{i}$ is the nominal value of the input for the i-th scenario of nominal points, presented in Section 3, and $-{\left(h_{D}\right)}_{i}$ is the respective transmission pole of the linear approximant. For demonstration purposes, two distinct values of a will be examined. The first is a=3.16309 and the second is a=4.74463, corresponding to $a=2\underset{i=\left\{1,\ldots ,10\right\}}{\max}\left\{U_{i},{\left(h_{D}\right)}_{i}\right\}$ and $a=3\underset{i=\left\{1,\ldots ,10\right\}}{\max}\left\{U_{i},{\left(h_{D}\right)}_{i}\right\}$, respectively. The threshold $\zeta_{O,A}$ is chosen to be $\zeta_{O,A}=0.5$. The optimal observer parameters for both cases of a and all scenarios of nominal points, given in Table S1 of Supplementary Material, are presented in Tables S2 and S3 of Supplementary Material, where the results show that, in all cases, $\rho_{F,1}=a+$, i.e., $\rho_{F,1}$ tends to a as $\rho_{F,1}$ decreases and $J_{e,A}=\zeta_{O,A}$. In Figures S1 and S2 of Supplementary Material, the regions satisfying the inequality constraint used during minimization, without considering ${\tilde{J}}_{e,O}$, are presented for all scenarios of nominal points. In particular, the frontiers of these regions are presented using different color curves for each scenario in a wide enough range of observer poles. The regions extend to the lower side of each curve. For both choices of a, mentioned in the previous paragraph, it is observed that there exists a wide range of valid observer poles. These regions can be used to in the derivation of a suboptimal solution of the observer parameters, resulting in competitively small ${\tilde{J}}_{e,O}$, as compared to the optimal solutions in Tables S2 and S3 of the Supplementary Material. These suboptimal solutions could be derived through a metaheuristic algorithm, indicatively see [37,38]. To demonstrate this characteristic, the minimum and maximum values of ${\tilde{J}}_{e,O}$ and $J_{e,A}$, for both choices of a, are presented in Table S4 and Table S5 of the Supplementary Material, where small differences between the maximum and the minimal values ${\tilde{J}}_{e,O}$ are observed.

## 5. A Framework for Linear Observer Design through Parameter Identification of SISO I/O Linear Approximants

### 5.1. The General Framework

The general linear time inariant state space approximant of a SISO process is in the form (4). The SISO system is in general nonlinear, i.e., it has the description:

(54) $$\dot{\tilde{x}}(t)=f_{NL}(\tilde{x}(t),u(t)),\;\;x(0-)=x_{0},\;y(t)=c_{NL}(\tilde{x}(t)),\;\tilde{x}\in {\mathbb{R}}^{n\times 1},y,u\in \mathbb{R},$$

where $\tilde{x}(t)$, u(t) and y(t) denote the vector of the state variables, the input variable, and the output variable of the nonlinear process model (54). The respective nominal values are denoted by $\tilde{X}$, U and Y, respectively. The variations of the system variables around the nominal values are denoted by $\Delta \tilde{x}(t)=\tilde{x}(t)-\tilde{X}$, Δy(t)=y(t)−Y and Δu(t)=u(t)−U, respectively. The system (54) is assumed to be globally stable and consequently the respective state space linear approximant is stable. Additionally, it is assumed that for every bounded input, with steady state value, the resulting responses of the state variables are bounded having steady state values. Regarding system (54), it is also assumed that

(a) The structure of the vector functions $f_{NL}(\cdot ,\cdot )$ is known but the physical parameters evaluating the elements of the vector function are not known except the parameters being independent of current characteristics of the process. Indicatively, for the case of the chemostat presented in Section 2, all parameters are unknown except the parameter δ.
(b) The output variable and the input variable of the process are measured in real time.
(c) Additionally, the operating trajectory of the nonlinear process, namely the values of $\tilde{X}$ and Y for every U, in an appropriate operation domain, is considered to be known. Indicatively see Figure 1 where the operating trajectory of a chemostat is depicted. The operating trajectory can be determined using small scale experiments around different operating values of the process and possibly appropriate measurement devices, in the case where the process is out of production mode. The operation domain of the operating values of the input is denoted by ${ℍ}_{U}$.

Regarding the state space linear approximant of the nonlinear process, the following assumptions are basic for the development of the present framework.

**Assumption** **1.** *The structure of the system matrices of the linear approximant (4) are considered to have a known structure depending upon a set of physical parameters being independent among themselves and considered to be unknown to the designer and grouped in vector form as* $p=\left[\begin{array}{ccc} p_{1} & \cdots & p_{k} \end{array}\right]\in {\mathbb{R}}^{1\times r}\;$*, in other words, the system matrices are known functions of the unknown vector* p, i.e., $A=A(p)\in {\mathbb{R}}^{n\times n}$, $b=b(p)\in {\mathbb{R}}^{n\times 1}$, $c=c(p)\in {\mathbb{R}}^{1\times n}$.

**Remark** **1.** *Except the dependence upon the physical parameters, the system matrices depend also upon the respective operating point* ℓ=(Y,U)*, i.e.,*A=A(p,ℓ)*,*b=b(p,ℓ)*and*c=c(p,ℓ)*. The operating point belongs to a set of admissible operating points, denoted by*${ℍ}_{L}$*. This set is determined by technical characteristics of the process. The set of the admissible unknown system parameters is denoted by*${ℍ}_{p}\subseteq {\mathbb{R}}^{1\times r}$.

**Remark** **2.** *In general, the set of the elements of the vecto p is a subset of the complete set of physical parameters of the nonlinear process*.

**Assumption** **2.** *The linear approximant of the nonlinear process (54), with system matrices in the form* A=A(p,ℓ), b=b(p,ℓ)*and* c=c(p,ℓ)*, is observable* $\forall \ell \in {ℍ}_{L}$*and* $\forall p\in {ℍ}_{p}\subseteq {\mathbb{R}}^{1\times r}$.

The I/O approximant of the state space linear approximant system (4), in the general form, is

(55) $$S:\;\Delta y_{L}^{\left(n_{c}\right)}(t)+\left[\begin{array}{ccc} h_{D,1} & \cdots & h_{D,n_{c}} \end{array}\right]{\left[\begin{array}{ccc} \Delta y_{L}^{\left(n_{c}-1\right)}(t) & \cdots & \Delta y_{L}^{\left(0\right)}(t) \end{array}\right]}^{T}=\left[\begin{array}{ccc} h_{N,1} & \cdots & h_{N,n_{c}} \end{array}\right]{\left[\begin{array}{ccc} \Delta u^{\left(n_{c}-1\right)}(t) & \cdots & \Delta u^{\left(0\right)}(t) \end{array}\right]}^{T}.$$

where $h_{D,j}$ and $h_{N,j}$ are the real coefficients of the I/O approximant. Since the respective state space linear approximant is observable, the nonnegative integer $n_{c}$ is equal to the rank of the controllability matrix of the linear approximant, i.e., it is equal to the dimension of the controllable subsystem of the state space linear approximant. Define

(56) $$h=\left[\begin{array}{ccccccc} h_{D,1} & \cdots & h_{D,n_{c}} & | & h_{N,1} & \cdots & h_{N,n_{c}} \end{array}\right]\;\in {\mathbb{R}}^{1\times 2n_{c}}.$$

Considering the formulas A=A(p,ℓ),  b=b(p,ℓ), c=c(p,ℓ) and the formula of the transfer function of a linear time invariant system, the set of all admissible h, being a subset of ${\mathbb{R}}^{1\times 2n_{c}}$ and denoted by ℍ, is defined.

**Definition** **1.** *The state space linear approximant (4) is I/O reconstructable by the I/O linear approximant, if for every* h∈ℍ*and every for every* $\ell \in {ℍ}_{L}$*the system matrices* A=A(p,ℓ),  b=b(p,ℓ), c=c(p,ℓ)*are uniquely determined.*

**Assumption** **3.** *The class of nonlinear processes studied, here, is that of systems with I/O reconstructable state space linear approximants by the respective I/O linear approximants.*

From the knowledge of the operating trajectory, it is concluded that for every operating value of the input, there are unique and known operating values of the state variables and consequently there is a unique and known operating value of the measurement output. Thus, using Assumption 3, the system matrices of the state space linear approximant (4) can be expressed as follows:

(57) $$A=A(h,U)\in {\mathbb{R}}^{n\times n},\;\;b=b(h,U)\in {\mathbb{R}}^{n\times 1},\;\;c=c(h,U)\in {\mathbb{R}}^{1\times n};\;h\in ℍ,\;U\in {ℍ}_{U}.$$

**Remark** **3.** *For the state space linear approximant to be reconstructable it is necessary for the uncontrollable part of the system to be uniquely determined* ∀h∈ℍ*and*$\forall U\in {ℍ}_{U}$.

**Assumption** **4.** *The state space linear approximant (4), with system matrices in the form (57), is observable and stable* ∀h∈ℍ*and*$\forall U\in {ℍ}_{U}$.

Using Assumptions 3 and 4 and the general state space linear approximant with system matrices in the form (57), the general full order description in (21–23) is used, where the observer matrices are of the form

(58) $$F=F(h,U)\in {\mathbb{R}}^{n\times n},\;\;m=m(h,U)=b(h,U)\in {\mathbb{R}}^{n\times 1},\;g=g(h,U)\in {\mathbb{R}}^{1\times n};\;h\in ℍ,\;U\in {ℍ}_{U}.$$

Clearly, using (58), the dynamics of the observer estimation error in (25) are still valid in the present general case. 

In the case, where the coefficients of the I/O approximant (55) are determined using an identification algorithm, the following I/O linear model is used:

(59) $$S_{I}:\;\Delta y_{}^{\left(n_{c}\right)}(t)+\left[\begin{array}{ccc} {\hat{h}}_{D,1} & \cdots & {\hat{h}}_{D,n_{c}} \end{array}\right]{\left[\begin{array}{ccc} \Delta y_{}^{\left(n_{c}-1\right)}(t) & \cdots & \Delta y_{}^{\left(0\right)}(t) \end{array}\right]}^{T}=\Delta y_{}^{\left(n_{c}\right)}(t)+\left[\begin{array}{ccc} {\hat{h}}_{D,1} & \cdots & {\hat{h}}_{D,n_{c}} \end{array}\right]{\left[\begin{array}{ccc} \Delta y_{}^{\left(n_{c}-1\right)}(t) & \cdots & \Delta y_{}^{\left(0\right)}(t) \end{array}\right]}^{T}$$

where ${\hat{h}}_{D,j}$ and ${\hat{h}}_{N,j}$ are the identified parameters, being grouped into the following vector

(60) $$\hat{h}=\left[\begin{array}{ccccccc} {\hat{h}}_{D,1} & \cdots & {\hat{h}}_{D,n_{c}} & | & {\hat{h}}_{N,1} & \cdots & {\hat{h}}_{N,n_{c}} \end{array}\right]\;\in {\mathbb{R}}^{1\times 2n_{c}}$$

and where $\varepsilon_{y}(t)$ is the identification error in (59). In general, the identifications algorithms are based on the minimization of a norm of the modelling error (indicatively see [39,40]). For $\hat{h}$ to belong to ℍ, a slight modification of the identification algorithm is proposed. In particular, if the computed $\hat{h}$ is outside ℍ, then it is substituted by the nearest, or an near enough, vector in ℍ. Hence, in the case of identified coefficients and using (60), the observer matrices in (58) take on the forms

(61) $$F=F(\hat{h},U)\in {\mathbb{R}}^{n\times n},\;\;m=m(\hat{h},U)\in {\mathbb{R}}^{n\times 1},\;g=g(\hat{h},U)\in {\mathbb{R}}^{1\times n};\;\hat{h}\in ℍ,\;U\in {ℍ}_{U}.$$

In the present case, where only $\hat{h}$, U and Y are assumed to be known, the observer is proposed to be of the following full order form

(62) $$ℑ:\Delta \dot{z}(t)=F(\hat{h},U)\Delta z(t)+g(\hat{h},U)\Delta y(t)+m(\hat{h},U)\Delta u(t),\;\;\;\Delta z(0-)=\Delta z_{0}.$$

The goal of the above observer is to provide a response Δz approximating $\Delta \tilde{x}$, i.e., to obtain a small enough estimation error. The estimation error is defined to be

(63) $$e_{O}(t)=\Delta \tilde{x}(t)-\Delta z(t).$$

According to Assumption 2 and using $\hat{h}$, the system matrices of the linear approximant can be computed to be of the form

(64) $$A=A(\hat{h},U)\in {\mathbb{R}}^{n\times n},\;\;b=b(\hat{h},U)\in {\mathbb{R}}^{n\times 1},\;\;c=c(\hat{h},U)\in {\mathbb{R}}^{1\times n};\;\hat{h}\in ℍ,\;U\in {ℍ}_{U}.$$

The first and the second system matrices in (61) are computed in terms of (64) as follows:

(65) $$F(\hat{h},U)=A(\hat{h},U)-g(\hat{h},U)c(\hat{h},U)\in {\mathbb{R}}^{n\times n},\;m(\hat{h},U)=b(\hat{h},U).$$

**Remark** **4.** *From Assumption 4 and relation (65), it is observed that the eigenvalues of* $F(\hat{h},U)$*can arbitrarily be chosen through* $g(\hat{h},U)$*. Moreover, it is important to point out that the initial condition of the general observer form in (21)–(23) with system matrices in the form (58) is benefited as follows:* $c(\hat{h},U)\Delta z(0-)=y(0-)$*and consequently,* $c(\hat{h},U)e_{O}(0-)=0$.

Using Remark 4, the following design requirement is introduced. 

*Design requirement 1:* Using $g(\hat{h},U)$, the eigenvalues of $F(\hat{h},U)=$ $A(\hat{h},U)-g(\hat{h},U)c(\hat{h},U)$ are chosen to be a-regional stable, real and distinct, i.e.,

(66) $$0<a<\rho_{F,1}(\hat{h},U)<\cdots <\rho_{F,n}(\hat{h},U);\;\rho_{F,k}(\hat{h},U)\in {\mathbb{R}}_{+}$$

where $-\rho_{F,k}(\hat{h},U)$, k∈{1,...,n}, are the eigenvalues of $F(\hat{h},U)$ and where the eigenvalues in (66) are presented in ordered form. 

From the Design requirement 1, it is observed that the system matrices in (65) depend also upon the positive real number a, i.e.,

(67) $$F=F(\hat{h},U,a)=A(\hat{h},U)-g(\hat{h},U,a)c(\hat{h},U),\;g=g(\hat{h},U,a),m(\hat{h},U)=b(\hat{h},U),\rho_{F,k}=\rho_{F,k}(\hat{h},U,a).$$

The variations of the system variables, presented just after (54), satisfy the state space linear approximant with a respective modelling error, denoted by $\varepsilon_{x}(t)$. This modelling error satisfies the equation

(68) $$\Delta \dot{\tilde{x}}(t)=A(\hat{h},U)\Delta \tilde{x}(t)+b(\hat{h},U)\Delta u(t)+\varepsilon_{x}(t),\;\;\;\Delta \tilde{x}(0-)=\tilde{x}(0-)-\tilde{X}.$$

Clearly, it holds that $\varepsilon_{x}(0-)=0$.

At this point, the present framework is further specialized by the following two assumptions.

**Assumption** **5.** *The map of the vector of the state variables, of the nonlinear process, to the output variable is linear, i.e.,* $c_{NL}(\tilde{x})=c\tilde{x},\;c\in {\mathbb{R}}^{1\times n}$.

**Assumption** **6.** *The output matrix of the linear approximant is independent from the coefficients of the I/O linear approximant, i.e.,*c(h,U)=c.

From Assumptions 5 and 6, it is observed that

(69) $$c(h,U)=c(\hat{h},U)=c.$$

The following proposition, relating to the estimation error and the modelling error, is of particular importance.

**Proposition** **1.** *The observer estimation error based on the I/O linear approximant identified parameters is forced by the state space linear approximant modelling error, as follows:*

(70) $${\dot{e}}_{O}(t)=F(\hat{h},U,a)e_{O}(t)+\varepsilon_{x}(t),\;\;\;e_{O}(0-)=e_{O,0}.$$

**Proof** **of** **Proposition** **1.** From (62), (63), (67)–(69) and appropriate algebraic manipulations, the dynamic description (70) is derived.  □

Clearly, the response of (70) can be analyzed in two terms as follows:

(71) $$e_{O}(t)=e_{O,A}(t)+e_{O,B}(t),$$

where

(72a) $${\dot{e}}_{O,A}(t)=F(\hat{h},U,a)e_{O,A}(t),\;\;\;e_{O,A}(0-)=e_{O,0},$$

(72b) $${\dot{e}}_{O,B}(t)=F(\hat{h},U,a)e_{O,B}(t)+\varepsilon_{x}(t),\;\;\;e_{O,B}(0-)=0_{n\times 1}.$$

From Design requirement 1, it is observed that the response of (72a) can be expressed as follows

(73) $$e_{O,A}(t)=\left[\sum_{k=1}^{n}\exp\left(-\rho_{F,k}(\hat{h},U,a)t\right)\Phi_{k}(\hat{h},U,a)\right]e_{O,0};\;\;\Phi_{k}(\hat{h},U,a)\in {\mathbb{R}}^{n\times n},$$

where $-\rho_{F,k}(\hat{h},U,a)$ are the real, distinct and enough negative eigenvalues of $F(\hat{h},U,a)$ and $\Phi \left(t\right)=\sum_{k=1}^{n}\exp\left(-\rho_{F,k}(\hat{h},U,a)t\right)\Phi_{k}(\hat{h},U,a)$ is the transition matrix of system (72a), namely the inverse Laplace transform of the resolvent matrix ${\left(sI_{n}-F(\hat{h},U,a)\right)}^{-1}$. The coefficient matrices are determined to be

$$\Phi_{k}(\hat{h},U,a,\rho_{F}(\hat{h},U,a))=\underset{s\to \rho_{F,k}(\hat{h},U)}{\lim}\left[\left(s+\rho_{F,k}(\hat{h},U,a)\right){\left(sI_{n}-F(\hat{h},U,a,\rho_{F}(\hat{h},U,a))\right)}^{-1}\right],$$

where

(74) $$\rho_{F}(\hat{h},U,a)={\left[\begin{array}{ccc} \rho_{F,1}(\hat{h},U,a) & \cdots & \rho_{F,n}(\hat{h},U,a) \end{array}\right]}^{T}.$$

Regarding the eigenvalues of $F(\hat{h},U)$, it holds that

(75) $$\rho_{F,k}(\hat{h},U,a)>a,\;\;\forall k\in \{1,...,n\}.$$

From (73)–(75), it is observed that

(76) $${\left\|e_{O,A}(t)\right\|}_{\alpha }<\exp\left(-at\right)\left[{\sum_{k=1}^{n}\left\|\Phi_{k}(\hat{h},U,a,\rho_{F}(\hat{h},U,a))\right\|}_{\alpha }\right]{\left\|e_{O,0}\right\|}_{a},\;\forall t\geq 0,$$

where ${\left\|\cdot \right\|}_{\alpha }$ denotes the α norm of the argument vector or matrix and where α∈{1,2,...,∞}. From (76), it is observed that the rate of convergence of $e_{O,A}(t)$ depends upon a.

To guarantee that ${\left\|e_{O,A}(t)\right\|}_{\alpha }$ is enough small with respect to the initial condition of the estimation error, the following design requirement is imposed.

*Design requirement 2:* The design requirement is: find $\rho_{F}$ such that the following inequality is satisfied

(77) $$J_{e,A}^{*}(\hat{h},U,a,\rho_{F})=\exp\left(-a\right)\left[{\sum_{k=1}^{n}\left\|\Phi_{k}(\hat{h},U,a,\rho_{F})\right\|}_{\alpha }\right]\leq \zeta_{O,A},$$

where $\zeta_{O,A}\in {\mathbb{R}}_{+}$ is a small enough positive real number set by the designer. Since $\rho_{F}$ can be chosen arbitrarily, subject to the Design requirement 1, they are the degrees of freedom of the present design requirement.

**Remark** **5.** *The inequality (77) denotes an upper bound of the scale of the response at the critical time instant* t=1*. For (77) to be satisfied it is necessary for* ${\sum_{k=1}^{n}\left\|\Phi_{k}(\hat{h},U,a)\right\|}_{\alpha }$*to be exponentially bounded with respect to* $a\in {\mathbb{R}}_{+}$*. It is noted that in most processes the quantity*${\sum_{k=1}^{n}\left\|\Phi_{k}(\hat{h},U,a)\right\|}_{\alpha }$*is of rational form with respect to* a*. In these processes, for every* $\zeta_{O,A}$*, there exist a sufficiency large* a*guarantees (77).*

For (72b), the case of stepwise responses, namely the input signal is of the form $u(t)=u_{w}u_{s}(t)+U$, where $u_{s}(t)$ is the unitary step signal. Step wise transitions appear to be the most common type of transitions in industrial processes. Additionally, as already mentioned in the beginning of the section, the nonlinear process is assumed to be stable. From Assumption 4 and the property that $\hat{h}$ belongs to ℍ, it is concluded that the state space linear approximant, derived using the identified coefficients of the I/O linear approximant, is also stable. Finally, as already mentioned in the beginning of the section, the operating trajectory of the state variables of the process is known. Hence, through the operating trajectory, for a step wise command, the resulting vector of nominal values of the state variables is known and is denoted by ${\tilde{X}}_{w}$. The respective nominal value of the output variable is denoted by $y_{w}$ and is equal to $c{\tilde{X}}_{w}$. Using (68), the steady state value of the model error is computed to be

(78) $$\varepsilon_{x,SS}(\hat{h},U,u_{w})=\underset{t\to +\infty }{\lim}\;\varepsilon_{x}(t)=-A(\hat{h},U)({\tilde{X}}_{w}-\tilde{X})-b(\hat{h},U)u_{w}.$$

It is important to mention that the known vectors ${\tilde{X}}_{w}$ and $\tilde{X}$ are expressed in the form ${\tilde{X}}_{w}={\tilde{X}}_{w}(U+u_{w})$ and $\tilde{X}=\tilde{X}(U)$. 

As already mentioned, using $g(\hat{h},U,a)$, the eigenvalues of $F(\hat{h},U,a)$ can be derived to be a-stable, real, and distinct. The vector $\rho_{F}(\hat{h},U,a)$ is the degree of freedom for the present design scheme. So, in what follows, it can be considered as the source for the determination of $g(\hat{h},U,a)$. So, we may write $g(\hat{h},U,a)=g(\hat{h},U,a,\rho_{F})$ and consequently $F(\hat{h},U,a,\rho_{F})$. Hence, using (71), (72) and (78), the steady state estimation error is computed to be

(79) $$e_{O,SS}(\hat{h},U,u_{w},a)={\left(A(\hat{h},U)-g(\hat{h},U,a,\rho_{F})c\right)}^{-1}\varepsilon_{x,SS}(\hat{h},U,u_{w}).$$

The following design requirement is introduced. 

*Design requirement 3:* The design goal is to minimize the ratio of the steady state estimation error to the steady state of the variation of the state vector, i.e.,

(80) $$\begin{array}{l} J_{e,O}^{*}(\hat{h},U,a,u_{w})=\underset{\rho_{F}}{\min}\left\{\frac{{\left(e_{O,SS}(\hat{h},U,a,u_{w})\right)}^{T}e_{O,SS}(\hat{h},U,a,u_{w})}{{\left({\tilde{X}}_{w}-\tilde{X}\right)}^{T}\left({\tilde{X}}_{w}-\tilde{X}\right)}\right\}=\\ \underset{\rho_{F}}{\min}\left\{{\left[{\left({\tilde{X}}_{w}-\tilde{X}\right)}^{T}\left({\tilde{X}}_{w}-\tilde{X}\right)\right]}^{-1}\varepsilon_{x,SS}^{T}(\hat{h},U,u_{w}){\left[\left(A(\hat{h},U)-g(\hat{h},U,a,\rho_{F})c\right)\left(A^{T}(\hat{h},U)-c^{T}g^{T}(\hat{h},U,\rho_{F})\right)\right]}^{-1}\varepsilon_{x,SS}(\hat{h},U,u_{w})\right\}=\\ \underset{\rho_{F}}{\min}\left\{{\left[{\left({\tilde{X}}_{w}-\tilde{X}\right)}^{T}\left({\tilde{X}}_{w}-\tilde{X}\right)\right]}^{-1}\left({\left({\tilde{X}}_{w}-\tilde{X}\right)}^{T}A^{T}(\hat{h},U)+u_{w}^{T}b^{T}(\hat{h},U)\right)\left[\left(A(\hat{h},U)-g(\hat{h},U,a,\rho_{F})c\right)\right.\right.=\\ \left.{\left.\left(A^{T}(\hat{h},U)-c^{T}g^{T}(\hat{h},U,\rho_{F})\right)\right]}^{-1}\left(A(\hat{h},U)({\tilde{X}}_{w}-\tilde{X})+b(\hat{h},U)u_{w}\right)\right\} \end{array}$$

subject to the constraints of Design requirement 1 and 2.

**Remark** **6.** *To further investigate (80), we define the following set, based on step responses of the nonlinear process,*

(81) $$T_{e,O}(\hat{h},U,a,\chi_{O})=\left\{u_{w}\in \mathbb{R}:J_{e,O}^{*}(\hat{h},U,a,u_{w})\leq \chi_{O}\;;\;u(t)=u_{w}u_{s}(t)+U\right\}.$$

**Remark** **7.** *The present observer design problem, defined for step responses of the nonlinear process, consists of determining the larger possible area* $T_{e,O}(\hat{h},U,a,\chi_{O,B})$*, for specific* $F(\hat{h},U,a,g)$*, determined by a specific* $g(\hat{h},U,a,\rho_{F})$*which in turn is determined by the vector* $\rho_{F}$*, that minimizes (80) subject to the constraints (66), as well as a specific* $\chi_{O}\in {\mathbb{R}}_{+}$*set by the designer.*

**Remark** **8.** *The solution of the cost minimization under constraints, presented above, can be derived either analytically or using a metaheuristic algorithm (indicatively, see [17,18,19,20]).*

### 5.2. Observer Design Using Parameter Identification of the I/O Linear Approximant of the Chemostat

In this subsection, the two coefficients of the I/O linear approximant in (6) are derived, using a parameter identification algorithm (indicatively see [39,40]). The parameter identification algorithm is driven by experimental measurement data of the deviations of the inputs and the outputs (I/O measurements), namely the variables y(t) and u(t), around an operating point (Y,U). Clearly, U is known and Y can be known through experimentation. Hence, Δy(t) and Δu(t) are directly derived. The I/O linear model used for identification, around the operating point (Y,U), is the following specification of (59)

(82) $$S_{I}:\;\;\;\Delta y^{\left(1\right)}(t)+{\hat{h}}_{D}\Delta y(t)={\hat{h}}_{N}\Delta u(t)+\varepsilon_{y}(t).$$

where ${\hat{h}}_{D}$ and ${\hat{h}}_{N}$ are the estimated (identified) values of the I/O linear approximant coefficients and $\varepsilon_{y}(t)$ is the respective identification error. Substituting, the identified values of the coefficients of the I/O linear approximant to the observer matrices, it can readily be verified that

(83a) $$F({\hat{h}}_{D},g_{1},g_{2})=\left[\begin{array}{cc} -{\hat{h}}_{D}-U-g_{1} & -U/\delta \\ {\hat{h}}_{D}\delta -g_{2} & 0 \end{array}\right],\;m(h_{n})=\left[\begin{array}{c} {\hat{h}}_{N}\\ -\delta {\hat{h}}_{N} \end{array}\right].$$

Following (37) and (39), the measurement output gains of the observer take on the form

(83b) $$g_{1}=2a-{\hat{h}}_{D}-U+\gamma_{1},\;g_{2}=\delta \left[{\hat{h}}_{D}-\frac{a}{U}\left(a+\gamma_{1}\right)-\gamma_{2}\right].$$

The matrix F and the coefficients of its characteristic polynomial, being independent of the identified coefficients, are given by (26) and (28b), respectively, remaining unaffected as in relation (40) and (41). Using (50), the alternative expressions of F in (51) can be used. Additionally, the formulas of the cost functions in (77) and (80) are those in (52) and (53) after substituting $h_{D}$ and $h_{N}$ with ${\hat{h}}_{D}$ and ${\hat{h}}_{N}$, respectively. 

In what follows, the least square procedure in [39] will be applied to identify ${\hat{h}}_{D}$ and ${\hat{h}}_{N}$ for each scenario of nominal points of the chemostat presented in Table S1 of the Supplementary Material. In all scenarios the system excitation is achieved by input signals in the form

(84) $$u(t)=U_{i}+\lambda_{i}f_{w}(t)\;\;;\;i=1,\ldots ,10,$$

where $U_{i}$ is the nominal value of the input for nominal operating point scenario i, $\lambda_{i}$ is a real scaling factor and $f_{w}(t)$ is a signal, reach enough to highlight the dynamics of the nonlinear system. The scaling factor $\lambda_{i}$ will be chosen to be equal to 0.5% of the maximum acceptable deviation of the input from the nominal value $U_{i}$. The maximum acceptable deviation is derived by checking the accuracy of the respective linear approximant, presented in Section 3. The continuous time signal $f_{w}(t)$ is generated using a pseudo-random real number generator between −1 and +1 and an appropriate low pass filter to derive the smooth continuous curve. The form of $f_{w}(t)$ is presented in Figure S3 of the Supplementary Material. For the identification procedure to be more realistic, an additive measurement noise is considered to be applied. The noise signal is of fast varying continuous time random type of the form partly presented in Figure S4 of the Supplementary Material. For the implementation of the identification algorithm, a low pass Butterworth filter will be employed. The filter is designed setting the passband frequency at $1\left[\mathrm{rad}/s\right]$, the stopband frequency at $1.1\left[\mathrm{rad}/s\right]$ and the respective attenuations at 1 and 40, respectively. The Bode plot of the produced filter is presented in Figure S5 of Supplementary Material.

In all scenarios, the initialization of the identification algorithm will be accomplished by setting the initial value of the inverse correlation matrix to be equal to $10^{4}I_{2}$, where $I_{2}$ is the two-by-two identity matrix, while the initial estimations of all identified parameters will be selected to be equal to zero. It is important to mention that in all cases, the estimations of the unknown parameters, after a small period, slightly oscillate around constant values (indicatively see Figures S6 and S7 of Supplementary Material). The final estimate of each parameter is computed to be the average of the signal of each parameter estimation. In all scenarios, the integrals for the determination of the average are evaluated from $t=200\left[\mathrm{days}\right]$ to $t=400\left[\mathrm{days}\right]$. In Table S6 of the Supplementary Material, the identification results are presented for all scenarios of nominal points. Additionally, in Table S6 of Supplementary Material, the percentile fluctuation of the estimation signal of each estimated variable, around its average, and the true value of the unknown parameter, derived from the respective linear approximant are also presented. It can readily be verified that the identified values of the I/O coefficients are relatively accurate presenting small fluctuations.

Using the results of the above presented identification and using the observer parameters presented in Tables S2 and S3 of the Supplementary Material, the metric $J_{e,O}^{*}(\hat{h},U,a,u_{w})/u_{w}^{2}$ is also presented in Table S7 of the Supplementary Material The choice of the metric $J_{e,O}^{*}(\hat{h},U,a,u_{w})/u_{w}^{2}$ is based on the property that is independent from the values of $u_{w}$. Furthermore, it is mentioned that the metric $J_{e,A}^{*}(\hat{h},U,a,\rho_{F})$ for α=2 is given by (43). It is observed that this metric does not depend upon ${\hat{h}}_{D}$ and ${\hat{h}}_{N}$. So, its value is equal to 0.5, presented in Tables S2 and S3 of Supplementary Material.

## 6. A Framework for Switching Observer Design through Parameter Identification of SISO I/O Linear Approximants

### 6.1. A Multi-Model Description of a Nonlinear SISO Process

Consider a SISO nonlinear process of the form (54), where y(t) is the measurement output variable, $\tilde{x}(t)\in {\mathbb{R}}^{n\times 1}$ is the state vector and u(t) is the input variable. Let $L=\left\{\ell_{1},\ell_{2},\ldots ,\ell_{\mu }\right\}$ be a set of nominal operating points of the process, where $\ell_{i}=(Y_{i},U_{i})$ with $Y_{i}$ and $U_{i}$ denoting the corresponding nominal output and input values and i∈{1,…,μ}. Around the nominal operating point $\ell_{i}$, the nonlinear process is approximated by the respective linear state space approximants ${ℵ}_{i}=(A_{i},b_{i},c)$, being in the general form (4), i.e.,

(85) $${ℵ}_{i}:\;\Delta_{i}{\dot{x}}_{L}(t)=A_{i}\Delta_{i}x_{L}(t)+b_{i}\Delta_{i}u(t),\;\Delta_{i}y_{L}(t)=c_{i}\Delta_{i}x_{L}(t),\Delta_{i}x_{L}(0-)=\Delta_{i}x_{L,0}={\tilde{x}}_{0}-{\tilde{X}}_{i},$$

where ${\tilde{X}}_{i}$ is the vector of the operating values of the state variables corresponding to the operating point $\ell_{i}$, and where $\Delta_{i}y_{L}(t)$ and $\Delta_{i}x_{L}(t)$ are the approximants of the deviations $\Delta_{i}y(t)=y(t)-Y_{i}$ and $\Delta_{i}\tilde{x}(t)=\tilde{x}(t)-{\tilde{X}}_{i}$, respectively. The input of the linear approximant is the deviations $\Delta_{i}u(t)=u(t)-U_{i}$.

**Assumption** **7.** *The dimensions of the controllable subsystems of all state space linear approximants* ${ℵ}_{i}$*are equal.*

Using Assumption 7, the respective I/O linear approximant is

(86) $$S_{i}:\;\Delta_{i}y_{L}^{\left(n_{c}\right)}(t)+\left[\begin{array}{ccc} h_{D,1} & \cdots & h_{D,n_{c}} \end{array}\right]{\left[\begin{array}{ccc} \Delta_{i}y_{L}^{\left(n_{c}-1\right)}(t) & \cdots & \Delta_{i}y_{L}^{\left(0\right)}(t) \end{array}\right]}^{T}=\left[\begin{array}{ccc} h_{i.N,1} & \cdots & h_{i,N,n_{c}} \end{array}\right]{\left[\begin{array}{ccc} \Delta_{i}u^{\left(n_{c}-1\right)}(t) & \cdots & \Delta_{i}u^{\left(0\right)}(t) \end{array}\right]}^{T}$$

where $h_{i,D,j}$ and $h_{i,N,j}$ are the real coefficients of the I/O approximant. The nonnegative integer $n_{c}$ is the same for all ${ℵ}_{i}$**.** Define

(87) $$h_{i}=\left[\begin{array}{ccccccc} h_{i,D,1} & \cdots & h_{i,D,n_{c}} & | & h_{i,N,1} & \cdots & h_{i,N,n_{c}} \end{array}\right]\;\in {\mathbb{R}}^{1\times 2n_{c}},i\in \{1,\ldots ,\mu \}.$$

Assumption 3 is considered to hold for all ${ℵ}_{i}$, i.e., the systems ${ℵ}_{i}$ are reconstructable by the coefficients of the respective I/O linear approximant $S_{i}$ and the respecting operating value of the input $U_{i}$. Thus, the system matrices of ${ℵ}_{i}$ are expressed following the form (57), i.e.,

(88) $$A_{i}=A_{i}(h_{i},U_{i})\in {\mathbb{R}}^{n\times n},\;\;b_{i}=b_{i}(h_{i},U_{i})\in {\mathbb{R}}^{n\times 1},\;\;c_{i}=c_{i}(h_{i},U_{i})\in {\mathbb{R}}^{1\times n};h_{i}\in ℍ,\;U_{i}\in {ℍ}_{U},i\in \{1,\ldots ,\mu \}$$

where $h_{i}\in {\mathbb{R}}^{1\times n_{c}}$, being the vector of the coefficients of $S_{i}$, describes the process around the operating point $\ell_{i}$. For this vector to be well defined, the following assumption is introduced.

**Assumption** **8.** *All state space linear approximants* ${ℵ}_{i}$*are observable and stable for* $h_{i}\in ℍ$*and*$U_{i}\in {ℍ}_{U}$,i∈{1,…,μ}.

### 6.2. Observer Design Using the Coefficients of Each I/O Linear Approximant of the Web of Operating Points

A bank of observers will be designed. The bank of observers includes the observers ${ℑ}_{L,1},...,{ℑ}_{L.\mu }$, where one observer is designed for the linear approximant ${ℵ}_{i}$. The observer ${ℑ}_{L,i}$, where i∈{1,…,μ} is in the form

(89) ℑL.i: Δix^˙L(t)=FiΔix^L(t)+giΔiyL(t)+miΔiu(t), Δix^L(0−)=Δix^L,0

where $\Delta_{i}{\hat{x}}_{L}(t)$ is the estimation of the state vector of the linear approximant ${ℵ}_{i}=(A_{i},b_{i},c)$ and the estimation error of ${ℑ}_{L,i}$ is

(90) eiL(t)=ΔixL(t)−Δix^L(t),i∈{1,…,μ}.

The estimation of the original state variable vector $\tilde{x}(t)$ is proposed to be

(91) x^i(t)=Δix^L(t)+X˜i,i∈{1,…,μ}.

The estimation error of the state vector $\tilde{x}(t)$ is

(92) $${\;}^{i}e(t)=\;\tilde{x}(t)-{\;}^{i}\hat{x}(t)=\Delta_{i}\tilde{x}(t)-\Delta_{i}{\hat{x}}_{L}(t);\;\Delta_{i}\tilde{x}(t)=\tilde{x}(t)-{\tilde{X}}_{i}.$$

The observer gain matrices, in terms of $h_{i}\in ℍ$ and $U_{i}\in {ℍ}_{U}$, are expressed as follows:

(93) Fi=Fi(hi,Ui)∈ℝn×n,  mi=mi(hi,Ui)=bi(hi,Ui)∈ℝn×1, gi= gi(hi,Ui)∈ℝ1×n;hi∈ℍ, Ui∈ℍU,i∈{1,…,μ}

The estimation error of the linear approximant is governed by the equation

(94) e˙Li(t)=Fi(hi,Ui)eiL(t),i∈{1,…,μ}.

The coefficients of the characteristic polynomial of Fi(hi,Ui) can arbitrarily be assigned using gi(hi,Ui). To achieve enough small estimation error, the requirement adopted here is regional stability of Fi. Consider the a− regional stability, i.e., that the eigenvalues of Fi must belong to ℂa−=s∈ℂ:Res<−a, where a is a non-negative real, i.e., $a\in {\mathbb{R}}_{0}^{+}=\{\alpha \in \mathbb{R}:\alpha \geq 0\}$. The class of gi(hi,Ui) satisfying this property can be expressed by a set of inequalities determined using the classical Routh–Hurwitz criterion. Additionally, it is required for the roots of Fi to be real and distinct. 

### 6.3. Observer Design Using the Identified Coefficients of Each I/O Linear Approximant of the Web of Operating Points

In the case where the coefficients of the I/O approximant (86) are determined using an identification algorithm, the following I/O linear model is also used

(95) SI,i: Δiy(nc)(t)+h^i,D,1⋯h^i,D,ncΔiy(nc−1)(t)⋯Δiy(0)(t)T=h^i,N,1⋯h^i,N,ncΔiu(nc−1)(t)⋯Δiu(0)(t)T+εiy(t)

where ${\hat{h}}_{i,D,j}$ and ${\hat{h}}_{i,N,j}$ are the identified parameters, being grouped to the following vector:

(96) $${\hat{h}}_{i}=\left[\begin{array}{ccccccc} {\hat{h}}_{i,D,1} & \cdots & {\hat{h}}_{i,D,n_{c}} & | & {\hat{h}}_{i,N,1} & \cdots & {\hat{h}}_{i,N,n_{c}} \end{array}\right]\;\in {\mathbb{R}}^{1\times 2n_{c}},i\in \{1,\ldots ,\mu \}.$$

For ${\hat{h}}_{i}$ to belong to ℍ, the already reported in Section 5 modification of the identification algorithm is used. Hence, in the case of identified coefficients and using (96), the observer matrices in (94) have the following forms:

(97) Fi=Fi(h^i,Ui)∈ℝn×n,  mi=mi(h^i,Ui)=bi(h^i,Ui)∈ℝn×1, gi= gi(h^i,Ui)∈ℝ1×nh^i∈ℍ, Ui∈ℍU,i∈{1,…,μ}.

The dynamic description of the respective observer is in the form

(98) ℑi:Δiz˙(t)=Fi(h^i,Ui)Δiz(t)+gi(h^i,Ui)Δiy(t)+mi(h^i,Ui)Δiu(t),   Δiz(t0−)=Δiz0,

where $t_{0}$ is the time instant when the observer starts to produce state estimation signals. Recall that $\Delta_{i}y=y-Y_{i}$. The goal for $\Delta_{i}z$ is to approximate $\Delta_{i}\tilde{x}=\tilde{x}-{\tilde{X}}_{i}$, i.e., to obtain a small estimation error. The estimation error is defined to be

(99) eiO(t)=Δix˜(t)−Δiz(t),i∈{1,…,μ}.

Clearly, the estimation error vector eiO(t) is governed by the equations

(100) e˙Oi(t)=Fi(h^i,Ui)eiO(t)+εix(t),   eiO(0−)=eiO,0, i∈{1,…,μ},

Where εix(t) is the respective modelling error, determined as follows:

(101) Δix˜˙(t)=Ai(h^i,Ui)Δix˜(t)+bi(h^i,Ui)Δiu(t)+εxi(t),   Δix˜(0−)=x˜(0−)−X˜i, i∈{1,…,μ}

**Remark** **9.** *Using appropriate* gi(h^i,Ui)*, the eigenvalues of* Fi(h^i,Ui)*are* ai−*regionally stable, distinct and real. In many processes, the stability margin* ai*can be set to be the same for all observers. However, in other processes it can be selected to depend upon the index of the respective operating point. The design of the present observers can be accomplished using the results of Section 5, namely the Design requirements 1, 2 and 3. In the present case, the eigenvalues of* Fi(h^i,Ui)*are denoted by* −ρFi(h^i,Ui,ai).

**Remark** **10.** *According to the above, a bank of observers for the nonlinear process has been designed. The bank of observers includes the observers* ${ℑ}_{1},...,{ℑ}_{\mu }$*. The bank of observers is orchestrated via an appropriate switching mechanism that will be presented in the following subsection. This bank of observers, together with the switching mechanism, is the soft sensor of the state variables of the nonlinear process.*

### 6.4. Stepwise Transitions

In this subsection, the case of step wise transitions will be analyzed. Consider a transition from an initial operating point to a destination operating point. The initial operating point is denoted by $\ell_{I}=(Y_{I},U_{I})$. The respective nominal value of the state vector is denoted by ${\tilde{X}}_{I}$. The destination point is denoted by $\ell_{D}=(Y_{D},U_{D})$. The respective nominal value of the state vector is denoted by ${\tilde{X}}_{D}$. Additionally, it is considered that the observer used during this transition is designed using the nominal operating point $\ell_{i}=(Y_{i},U_{i})$, where nominal value of the state vector is ${\tilde{X}}_{i}$, and the respective identified data ${\hat{h}}_{i}$. The observer matrices are in the form (97). In terms of the original input signal of the nonlinear process, the transition is accomplished using the command

(102) $$u(t)=U_{I}+(U_{D}-U_{I})u_{s}(t),\;i\in \{1,\ldots ,\mu \}.$$

In terms of the variation of the input from the nominal value $U_{i}$, the transition is accomplished using the command

(103) $$\Delta_{i}u(t)=(U_{I}-U_{i})+(U_{D}-U_{I})u_{s}(t),i\in \{1,\ldots ,\mu \}.$$

In the present case, the Design requirements 1 and 2 are preserved and expressed as follows: 

*Design requirement 4:* The eigenvalues of Fi(h^i,Ui)=Ai(h^i,Ui)−gi(h^i,Ui)ci are chosen to be a-regional stable, real and distinct, i.e.,

(104) 0<ai<ρF,1i(h^i,Ui)<⋯<ρF,ni(h^i,Ui) ; ρF,ki(h^i,Ui)∈ℝ+,i∈{1,…,μ}.

Using the eigenvalues, the following inequality is required to be satisfied

(105) Jie,A*(h^i,Ui,ai,ρFi)=exp−ai∑k=1nΦki(h^i,Ui,ai,ρFi)α≤ζO,Ai,

where ζO,Ai∈ℝ+ is an enough small positive real number set by the designer, and where

(106) Φki(h^i,Ui,ai,ρFi)=lims→ρF,kis+ρF,kisIn−Fi(h^i,Ui,ai,ρFi)−1,ρFi=ρF,1i⋯ρF,niT,i∈{1,…,μ}.

Here, Design requirement 3, is appropriately modified to express the present multi observer case. In particular, the steady state value of the modelling error is computed to be

(107) εx,SSi(h^i,Ui,UD−Ui)=limt→+∞ εxi(t)=−Ai(h^i,Ui)(X˜D−X˜i)−bi(h^i,Ui)(UD−Ui).

The steady state estimation error is also computed to be

(108) eO,SSi(h^i,Ui,UD−Ui,ai)=Ai(h^i,Ui)−gi(h^i,Ui,ai,ρFi)ci−1εx,SSi(h^i,Ui,UD−Ui).

Thus, Design requirement 3 is now expressed as follows: 

*Design requirement 5:* The design goal is to minimize the ratio of the steady state estimation error to the steady state of the variation of the state vector, i.e.,

(109) Je,O∗i(h^i,Ui,ai,UD−Ui)=minρFeO,SSi(h^i,Ui,ai,UD−Ui)TeO,SSi(h^i,Ui,ai,UD−Ui)X˜w−X˜iTX˜w−X˜i=minρFiX˜D−X˜iTX˜D−X˜i−1(X˜D−X˜i)TAiT(h^i,Ui)+(UD−Ui)T(biT(h^i,Ui))Ai(h^i,Ui)−gi(h^i,Ui,ai,ρFi)ciAiT(h^i,Ui)−ciT(giT(h^i,Ui,ρFi))−1Ai(h^i,Ui)(X˜D−X˜)+bi(h^i,Ui)(UD−Ui)

subject to the constraints of Design requirement 4.

**Remark** **11.** *The following set of input nominal values, called target operating area and being a generalization of the set defined in Remark 8, is an interval around the operating point* $U_{i}$*, where any transition command with initial and destination command in this set satisfies Design requirements 4 and 5*

(110) T(Ui)=[Ui−ui,max,Ui+ui,max]⊆TOi(h^i,Ui,a,χO,A,χO,B);  ui,max=maxui∈ℝ+:[Ui−ui,Ui+ui]⊆TOi(h^i,Ui,a,χO,A,χO,B)

*where*

(111) TOi(h^i,Ui,a,χO,A,χO,B)=(UI,UD)∈ℝ×ℝ:Je,A∗i(h^i,Ui,ai,ρFi)≤χO,A∧ Je,O∗i(h^i,Ui,ai,UD−Ui)≤χO,B ;u(t)=(UD−Ui)us(t)+UI

The above target operating area has several differences as compared to the respective target operating areas, defined in [22,24], for the pure (without observer) control design problem. 

Without loss of generality, the elements of the set of the operating points are considered to be ordered in the sense that

(112) $$U_{i}<U_{i+1},\;\forall i\in \{1,\ldots ,\mu -1\},\;\mathrm{if}\;\mu >1.$$

In the present case, the dense web principle, first introduced in [22] is expressed as follows

(113) $$T(U_{i})\cap T(U_{i+1})\neq \emptyset ,\;\forall i\in \{1,\ldots ,\mu -1\},\;\mathrm{if}\;\mu >1.$$

Regarding the starting and the ending points of the intervals of two neighbor target operating areas, the condition (113) can be interpreted, as follows

(114) $$\max\left\{T(U_{i})\right\}<\min\left\{T(U_{i+1})\right\},\forall i\in \{1,\ldots ,\mu -1\},\;\mathrm{if}\;\mu >1.$$

**Remark** **12.** *The satisfaction of the dense web principle is a prerequisite for the observer design scheme to be satisfied. If the dense web principle is satisfied, or at least it is satisfied for a subset of adjacent operating points, the proposed switching observer scheme is of the stepwise safe switching type for the range of the operating trajectories covered by the union of the respective target operating areas.*

**Remark** **13.** *The respective step wise transitions are safe if the transition from one initial operating point* $\ell_{I}=(Y_{I},U_{I})$*to a destination operating point* $\ell_{D}=(Y_{D},U_{D})$*, where*$U_{I}\leq U_{D}$*, is divided to appropriate individual transitions. Let*$U_{I}\in T(U_{i})$*and* $U_{D}\in T(U_{i+\nu })$*, where* i∈{1,…,μ}*and* ν∈{0,…,μ−i}*. Let*

(115) $$\nu_{\sigma }=\max\left\{j\in \{i+\sigma -1,\ldots ,\nu \}:T(U_{i+\nu_{\sigma -1}})\cap T(U_{j})\neq \emptyset \right\},\;\sigma \in \{0,...,\nu \}$$

The first individual transition is from $\ell_{I}=(Y_{I},U_{I})$ to the intermediate destination point $\ell_{D,1}=(Y_{D,1},U_{D,1})$, where $U_{D,1}\in T(U_{i})\cap T(U_{i+\nu_{1}})$. The second individual transition is from $\ell_{D,1}=(Y_{D,1},U_{D,1})$, to the next intermediate $\ell_{D,2}=(Y_{D,2},U_{D,2})$, where $U_{D,2}\in T(U_{i+\nu_{1}})\cap T(U_{i+\nu_{2}})$. The individual transitions continue till the final destination point, namely the operating point $\ell_{D,f}=(Y_{D,f},U_{D,f})$, where $U_{D,f}\in T(U_{i+\nu_{f-1}})\cap T(U_{i+\nu_{f}})$, and $\nu_{f}=\nu$. Clearly, $\ell_{D,f}=\ell_{D}=(Y_{D},U_{D})$, while f is the total number of the intermediate transitions. It is obvious that the transition from $\ell_{D}=(Y_{D},U_{D})$ to $\ell_{I}=(Y_{I},U_{I})$ follows the reverse procedure.

We are now in a position to present the switching algorithm. During the stepwise multi step transition it is considered that system arrives at $\ell_{D,\sigma }=(Y_{D,\sigma },U_{D,\sigma })$ if it is very near to ${\tilde{X}}_{D,\sigma }$. Recall that the transitions are required to extend near the operating trajectory. The time, where the next transition is triggered, is when the system has approached $\ell_{D,\sigma }=(Y_{D,\sigma },U_{D,\sigma })$, when moving from $\ell_{D,\sigma -1}=(Y_{D,\sigma -1},U_{D,\sigma -1})$. This time is denoted by $\tau_{i+\nu_{\sigma -1}}^{*}$ and is chosen to be between $\tau_{i+\nu_{\sigma -1}}$ and $2\tau_{i+\nu_{\sigma -1}}$, where $\tau_{i+\nu_{\sigma -1}}$ is the settling time of the linear approximant $X_{i+\nu_{\sigma -1}}$. If $\tau_{i+\nu_{\sigma -1}}$ is not available to the designer, then the respective settling time of the I/O system behavior is used via small scale experimentation to the original nonlinear process. In what follows, this case will be considered to hold true. So, during the transition, the sequence of the observers is $J_{i},J_{i+\nu_{1}},...,J_{i+\nu_{f-1}}$. The sequence of time instants, when the different observers are applied, is $t_{0,}t_{0}+2\tau_{i}^{*},t_{0}+2\tau_{i+\nu_{1}}^{*},...,t_{0}+2\tau_{i+\nu_{f-1}}^{*}$, where $t_{0}$ is the time instant when the first intermediate transition is triggered. 

The reverse procedure, namely the transition from $\ell_{D}=(Y_{D},U_{D})$ to $\ell_{I}=(Y_{I},U_{I})$ is not symmetric, i.e., the transition sequence of the observers is $J_{i+\nu_{f}},J_{i+\nu_{f-1}},...,J_{i+\nu_{1}}$. The sequence of time instants, when the different observers are applied, is $t_{0},t_{0}+2\tau_{i+\nu_{f}}^{*},t_{0}+2\tau_{i+\nu_{f-1}}^{*},...t_{0}+2\tau_{i+\nu_{1}}^{*}$, where $t_{0}$ is the time instant when the first intermediate transition of the reverse procedure is triggered, and the respective observer starts to produce state estimation signals. 

Before closing this subsection, it is important to mention that upon switching the initial condition of the observer are the final conditions of the observer before the switching, i.e., in the direct procedure it holds that

(116) $$\Delta_{i}z(t_{0}-)={\tilde{X}}_{I},\;\Delta_{i+\nu_{\sigma }}z(t_{0}+2\tau_{i+\nu_{\sigma }}-)=\Delta_{i+\nu_{\sigma -1}}z(t_{0}+2\tau_{i+\nu_{\sigma -1}});\;\sigma =1,...,f.$$

while in the reverse procedure it holds that

(117) $$\Delta_{\nu }z(t_{0}-)={\tilde{X}}_{D},\;\Delta_{i+\nu_{\sigma -1}}z(t_{0}+2\tau_{i+\nu_{\sigma -1}}-)=\Delta_{i+\nu_{\sigma }}z(t_{0}+2\tau_{i+\nu_{\sigma }});\;\sigma =1,...,f.$$

**Remark** **14.** *In (110), the target areas have been defined to be symmetric intervals around* $U_{i}$*. The above analysis can also be valid for the case of nonsymmetric target areas. Indicative nonsymmetric target areas are presented in Section 6.5, including computational experiments for the process of the chemostat. However, for the unique determinations of nonsymmetric target areas, an algorithm with side extension priorities is required.*

**Remark** **15.** *The switching observers designed in this section as well as the respective stepwise transition depend greatly upon the choice of the set nominal operating values* $U_{i}$*, where* i∈{1,…,μ}*. In Section 6.5, different scenarios of operating points are investigated through computational experiments for the process of the chemostat.*

The soft sensor design, developed above, is analyzed to the following six basic steps:

Step 1: A set of operating points of the nonlinear process is determined. 

Step 2: Using small scale experiments around each operating point, the respective identified I/O linear approximants are determined. 

Step 3: Using the set of identified I/O linear approximants, if the reconstructability of the respective state space linear approximants is satisfied, then the respective state space linear approximants are computed. 

Step 4: Using the set of the state space linear approximants, the respective full order observers are computed, and the bank of observers is composed.

Step 5: Using the bank of observers and an estimation of the nonlinear process model, derived through an estimation of the physical parameters of the nonlinear model determined by the identified parameters of the I/O linear approximants, the target operating area of each operating point is determined.

Step 6: If the set of the target operating areas satisfies the dense web principle, then the transition through the switching algorithm is initialized, else go to Step 1 to determine a denser version of the set of the operating points. 

### 6.5. Performance of the Switching Observer Scheme for the Chemostat

In order to demonstrate the performance of the proposed switching observer scheme, consider the model parameters presented in Section 3. Furthermore, let a=3.5, $\chi_{O,A}=0.5$ and $\chi_{O,B}=0.001$. With respect to the target operating areas, two different cases for the nominal point sets will be examined. In the first case, the ten scenarios of nominal points presented in Section 3 will be used and the operating areas satisfying the constraints in (117) will be evaluated. Additionally, non-symmetric target operating areas will be considered to cover the entire area of valid nominal operating values of the input. The entire area is presented in (11b). In the second case, extensive computational experiments will be carried out to determine consecutive nominal points for the input with symmetric overlapping target operating areas.

**Figure 3:**
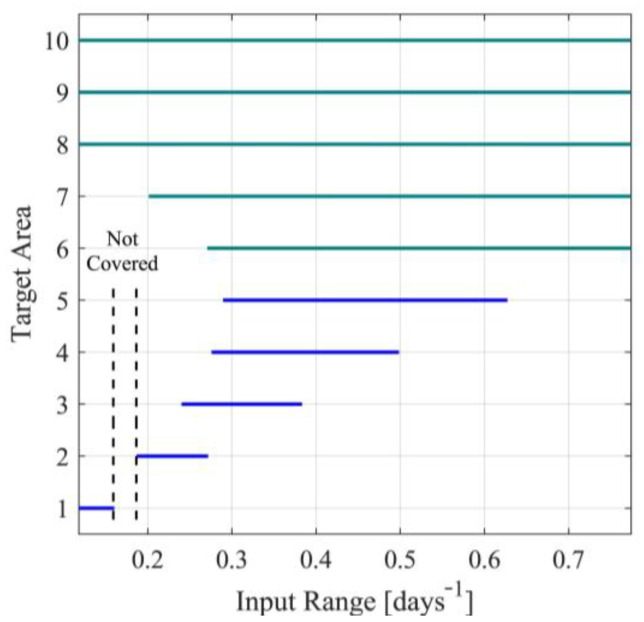
Target operating areas.

For the first case, note that the identified parameters ${\hat{h}}_{D}$ and ${\hat{h}}_{N}$, presented in Table S5 of Supplementary Material, will be used for each nominal value of the input $U_{i}$ (i=1,…,10), namely for each scenario. The parameters ρF,1i(h^i,Ui) and ρF,2i(h^i,Ui) will be derived by minimizing the metric in (115), under the inequality constraint for Je,A∗i. After the determination of the observer parameters, the target operating areas will also be determined. In Table S8 of Supplementary Material, for each nominal point $U_{i}$, the observer parameters $\rho_{F,1}$ and $\rho_{F,2}$, as well as the bounds $U_{i}-u_{i,\max}$ and $U_{i}+u_{i,\max}$ are presented. Note that the last column in Table S8 of the Supplementary Material is saturated, where needed, to $U_{\max}$ so that the inequality in (11c) is satisfied. Saturation has been performed for scenarios 6 to 10. This saturation results in nonsymmetric target operating areas since $U_{i}$ is no longer located at the center of the target operating area. The target operating areas are also graphically presented in Figure 3. From Table S8 of the Supplementary Material and Figure 3, it can also be observed that in the case of symmetric target operating areas, the area defined by $U_{D}\in \left(0.1603,0.1868\right)\left[{\mathrm{days}}^{-1}\right]$ is not covered by any operating area. Nevertheless, using any of the nonsymmetric cases 8, 9 or 10, that cover the entire area, this problem can be handled. 

In order to illustrate transitions between target operating areas, two simulation experiments will be carried out. In the first experiment, starting from an initial operating point $\ell_{I}=(Y_{I},U_{I})$ it is desirable for the system to settle to the final operating point $\ell_{D}=(Y_{D},U_{D})$, while passing through the intermediate destination points $\ell_{D,1}=(Y_{D,1},U_{D,1})$, $\ell_{D,2}=(Y_{D,2},U_{D,2})$ and $\ell_{D,3}=(Y_{D,3},U_{D,3})$. The point $\ell_{I}$, will be considered to correspond to target area 2. The points $\ell_{D,1}$ and $\ell_{D,2}$ will be considered to correspond to target areas 3 and 4, respectively. Finally, the points $\ell_{D,3}$ and $\ell_{D}$ will be considered to correspond to target area 5. The time, where each transition is triggered, in all cases, is chosen to be 10% greater than the settling time of the respective linear approximant. Let $Y_{I}=1.0709\left[{\mathrm{kg}/m}^{3}\right]$, $Y_{D,1}=1.3501\left[{\mathrm{kg}/m}^{3}\right]$, $Y_{D,2}=1.5247\left[{\mathrm{kg}/m}^{3}\right]$, $Y_{D,3}=2.9700\left[{\mathrm{kg}/m}^{3}\right]$, $Y_{D}=4.9500\left[{\mathrm{kg}/m}^{3}\right]$, $U_{I}=0.2134\left[{\mathrm{days}}^{-1}\right]$, $U_{D,1}=0.2579\left[{\mathrm{days}}^{-1}\right]$, $U_{D,2}=0.2826\left[{\mathrm{days}}^{-1}\right]$, $U_{D,3}=0.4500\left[{\mathrm{days}}^{-1}\right]$, $U_{D}=0.6000\left[{\mathrm{days}}^{-1}\right]$.

**Figure 4:**
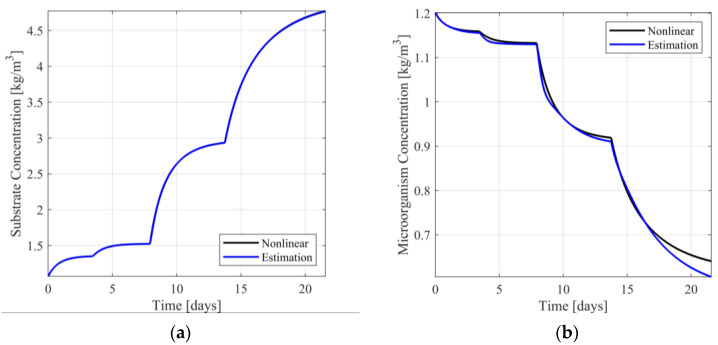
(**a**) Substrate concentration response/estimation; (**b**) Microorganism concentration response/estimation.

In Figure 4a,b, the responses of the nonlinear system and the switching observer scheme are presented. In Figures S8 and S9 of Supplementary Material the respective estimation errors are presented. From Figure 4a, it is observed that the estimation of the substrate concentration is visually identical to the respective nonlinear model response, presenting minimal estimation error (see Figure S8 of the Supplementary Material). Regarding the estimation of the microorganism concentration (see Figure 4b and Figure S9 of the Supplementary Material), the estimation is near the respective model response, presenting acceptable estimation error.

**Figure 5:**
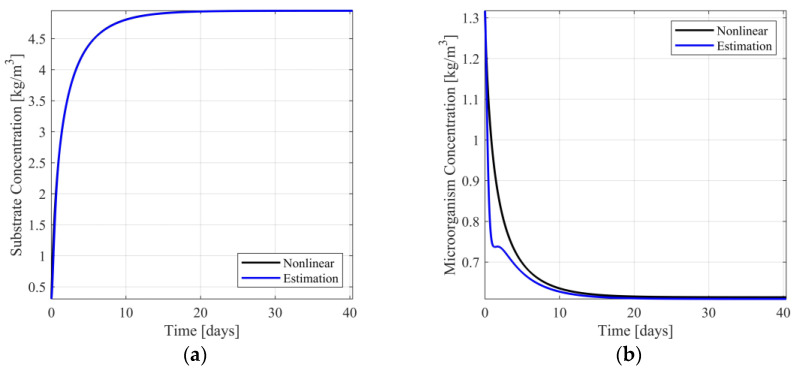
(**a**) Substrate concentration response and estimation for single step transition; (**b**) Microorganism concentration response and estimation for single step transition.

To simulate a transition from target area 1 to another operating point, being far from area 1, the use of one of the nonsymmetric target areas is necessary. Considering that scenarios 9 and 10 cover the entire area of nominal values of the input, transition between operating points can be carried out without any switching between observers, as the observer is designed upon the linear approximant corresponding either to scenario 9 or scenario 10. Indicatively, designing the observer using scenario 9 and simulating a single transition between $\ell_{I}=(Y_{I},U_{I})$ and $\ell_{D}=(Y_{D},U_{D})$, where $Y_{I}=0.3050\left[{\mathrm{kg}/m}^{3}\right]$, $Y_{D}=4.9500\left[{\mathrm{kg}/m}^{3}\right]$, $U_{I}=0.0697\left[{\mathrm{days}}^{-1}\right]$ and $U_{D}=0.6000\left[{\mathrm{days}}^{-1}\right]$, the responses of the nonlinear model, the observer, and the respective estimation error dynamics are presented in Figure 5, Figures S10 and S11 of Supplementary Material Similarly, to the previous computational experiment, the estimation of the substrate concentration (see Figure 5a) is visually identical to the respective nonlinear model response. The respective estimation error is minimal (see Figure S10 of Supplementary Material). Regarding the microorganism concentration, it is observed that although transition between operating points is faster and the steady state estimation error is small, the single step transition results in a transient estimation error being significantly larger than that of a multi-step approach (see Figure 5b and Figure S11 of the Supplementary Material).

**Figure 6:**
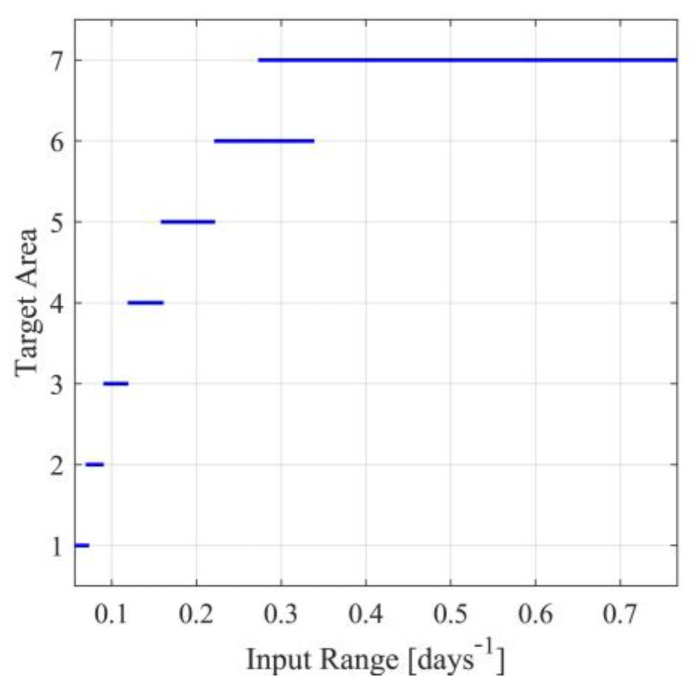
Target operating areas.

To avoid the presence of nominal points not being covered by a target area, a more extensive search approach will be implemented. Towards this aim, extensive computational experiments will be carried out to determine consecutive nominal points for the input with symmetric overlapping target operating areas. Let a=3.5, $\chi_{O,A}=0.5$ and $\chi_{O,B}=0.001$. A set of target operating areas satisfying the design requirements is presented in Table S9 of the Supplementary Material and Figure 6. In Table S9 of the Supplementary Material, besides the operating areas, the results of the identification procedure and the optimal observer parameters’ set for each target operating area, are also presented.

In order to illustrate transitions between target operating areas, starting from an initial operating point $\ell_{I}=(Y_{I},U_{I})$, it is desirable for the system to settle to the final operating point $\ell_{D}=(Y_{D},U_{D})$ passing through the intermediate destination points $\ell_{D,j}=(Y_{D,j},U_{D,j})$ (j=1,…,6). The point $\ell_{I}$ will be considered to correspond to target area 1. The points $\ell_{D,j}=(Y_{D,j},U_{D,j})$ (j=1,…,5) will be considered to correspond to target areas 2 to 6, respectively. Finally, the points $\ell_{D,6}$ and $\ell_{D}$ will be considered to correspond to target area 7. The time, where each transition is triggered, in all cases, is chosen to be 10% greater than the settling time of the respective linear approximant. Let

$Y_{I}=0.2642\left[{\mathrm{kg}/m}^{3}\right]$, $Y_{D,1}=0.3130\left[{\mathrm{kg}/m}^{3}\right]$, $Y_{D,2}=0.4036\left[{\mathrm{kg}/m}^{3}\right]$, $Y_{D,3}=0.5464\left[{\mathrm{kg}/m}^{3}\right]$, $Y_{D,4}=0.7589\left[{\mathrm{kg}/m}^{3}\right]$, $Y_{D,5}=\;1.1206\left[{\mathrm{kg}/m}^{3}\right]$, $Y_{D,6}=1.6938\left[{\mathrm{kg}/m}^{3}\right]$, $Y_{D}=3.7198\left[{\mathrm{kg}/m}^{3}\right]$, $U_{I}=0.0608\left[{\mathrm{days}}^{-1}\right]$, $U_{D,1}=0.0714\left[{\mathrm{days}}^{-1}\right]$, $U_{D,2}=0.0905\left[{\mathrm{days}}^{-1}\right]$, $U_{D,3}=0.1193\left[{\mathrm{days}}^{-1}\right]$, $U_{D,4}=0.1595\left[{\mathrm{days}}^{-1}\right]$, $U_{D,5}=0.2215\left[{\mathrm{days}}^{-1}\right]$, $U_{D,6}=0.3059\left[{\mathrm{days}}^{-1}\right]$, $U_{D}=0.5149\left[{\mathrm{days}}^{-1}\right]$.

**Figure 7:**
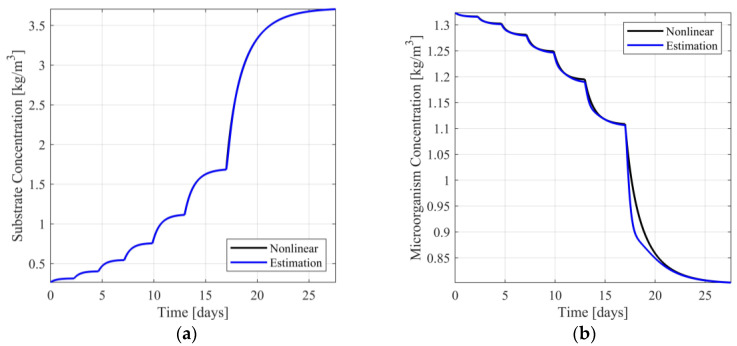
(**a**) Substrate concentration response and estimation; (**b**) Microorganism concentration response and estimation.

In Figure 7a,b, the response of the nonlinear system and the switching observer scheme are presented. In Figures S12 and S13 of the Supplementary Material, the respective estimation errors are presented. In accordance with the previous computational experiments, from Figure 7a it can readily be observed that the estimation of the substrate concentration is visually identical to the respective nonlinear model response, presenting minimal estimation error (see Figure S12 of Supplementary Material). With respect to the estimation of the microorganism concentration (see Figure 7b and Figure S13 of Supplementary Material), the estimation is near the respective model response, presenting acceptable estimation error, during the transient phase between target operating points.

## 7. Conclusions

A bank of full order linear switching observers has been designed for the development of soft sensors for the variables of single input single output (SISO) nonlinear processes. For the design of the bank of switching observer, a new switching design framework has been developed through the introduction of new definitions, system properties and results. A supervisor, orchestrating the switching among the observers, to approximate as close as possible the state variables of the nonlinear chemostat model, has been designed. A new set of target operating areas, oriented to observer design, has been introduced and the respective dense web principle for observer design has been introduced. Finally, the present design scheme has successfully been applied to the chemostat model.

An important aspect of the present results is that the design of the observer is based entirely upon the I/O linear approximant of the process model, being derived through standard I/O linear approximant coefficient identification using I/O data. Another important aspect is the derivation of the I/O data for both the identification and the operating trajectory can be derived using small scale experimentation. 

The algorithm for the realization of the soft sensor is simple and elegant, in the sense that it includes a bank of linear observer orchestrated by a simple supervisor rule. The proximity of the observed variables to respective real variables of the process is proven. Hence, the proposed algorithm is an adequate soft sensor for industrial processes. Overall, it is important to mention that the present results are offered for implementation to low-level computer platforms, such as μCs, PACs and other microprocessor embedded systems.

Before closing, it is important to mention that the full order form of the proposed here switching observers allows the detection of sudden increases of the estimation errors, corresponding to differences between the estimated and the original variables of the process, thus contributing to fault detection and fault isolation (see [41,42]). This is a first direction for future research. Another direction for future research is the extension of the present results to the category of multi-input and multi-output (MIMO) processes. This extension should take into account the vector form of the inputs and the outputs as well as the matrix forms of the I/O linear approximants and the observer matrices. Other directions are the extensions of the present results to other system categories. Particularly, the extension of the present results to singular systems process descriptions requires alternative observer design procedure (see [43,44]) and cost criteria handling the nonproper terms of the I/O linear approximants. The extension of the present results to the category of multi time delay systems (see [45,46]) requires the design of physically realizable observers as well as appropriate stability criteria.

## Data Availability

Data is contained within the article and supplementary material.

## Supplementary Materials

The following supporting information can be downloaded at: https://www.mdpi.com/article/10.3390/s23042114/s1, Table S1: Scenarios of nominal conditions; Table S2: Optimal observer parameter selection for a=3.16309; Table S3: Optimal observer parameter selection for a=4.74463; Table S4: Minimum and maximum metric values for a=3.16309; Table S5: Minimum and maximum metric values for a=4.74463; Table S6: Identification process results; Table S7: Identification error norms and steady state estimation error; Table S8: Observer parameter and target operating areas; Table S9: Observer parameter and target operating areas; Figure S1: Acceptable observer poles’ regions for a=3.16309 and all scenarios of nominal points; Figure S2: Acceptable observer poles’ regions for a=4.74463 and all scenarios of nominal points; Figure S3. Actuation signal $f_{w}(t)$; Figure S4: Additive measurement noise; Figure S5: Butterworth filter Bode plot; Figure S6: Estimation of $h_{D}$ for scenario 5; Figure S7: Estimation of $h_{N}$ for scenario 5; Figure S8: Substrate concentration estimation error; Figure S9: Microorganism concentration estimation error; Figure S10: Substrate concentration estimation error for single step transition; Figure S11: Microorganism concentration estimation error for single step transition; Figure S12: Substrate concentration estimation error; Figure S13: Microorganism concentration estimation error.

## References

[1] Liu Y., Xie M. (2020). Rebooting data-driven soft-sensors in process industries: A review of kernel methods. J. Process Control.

[2] Moushaee P., Babazadeh M. (2022). Pole assignment and distributed output feedback control via graph-based decomposition. Int. J. Control.

[3] Koumboulis F.N., Tzierakis K.G. (1998). Meeting transfer function requirements via static measurement output feedback. J. Frankl. Inst..

[4] Koumboulis F.N., Kouvakas N.D., Paraskevopoulos P.N. On the Morgan’s problem for neutral time delay systems via dynamic controllers with application to a test case central heating system. Proceedings of the 2009 IEEE Control Applications, (CCA) & Intelligent Control, (ISIC).

[5] Jiang Y., Yin S., Dong J., Kaynak O. (2021). A Review on Soft Sensors for Monitoring, Control and Optimization of Industrial Processes. IEEE Sens. J..

[6] Souza F.A.A., Araújo R., Mendes J. (2016). Review of soft sensor methods for regression application. Chemom. Intell. Lab. Syst..

[7] Ma M.-D., Ko J.-W., Wang S.-J., Wu M.-F., Jang S.-S., Shieh S.-S., Wong D.S.-H. (2009). Development of adaptive soft sensor based on statistical identification of key variables. Control Eng. Pract..

[8] Shoorehdeli M.A., Teshnehlab M., Sedigh A.K. (2009). Training ANFIS as an identifier with intelligent hybrid stable learning algorithm based on particle swarm optimization and extended Kalman filter. Fuzzy Sets Syst..

[9] Mendes J., Souza F., Araújo R., Gonçalves N. (2012). Genetic fuzzy system for data-driven soft sensors. Appl. Soft Comput..

[10] Li H., Gao Y., Shi P., Lam H. (2016). Observer-based fault detection for nonlinear systems with sensor fault and limited communication capacity. IEEE Trans. Autom. Control.

[11] Ding S.X. (2014). Data-driven design of monitoring and diagnosis systems for dynamic processes: A review of subspace technique based schemes and some recent results. J. Process Control.

[12] Yan W., Tang D., Lin Y. (2016). A data-driven soft sensor modeling method based on deep learning and its application. IEEE Trans. Ind. Electron..

[13] Wu X., Chen J., Xie L., Chan L.L.T., Chen C.-I. (2020). Development of convolutional neural network based gaussian process regression to construct a novel probabilistic virtual metrology in multi-stage semiconductor processes. Control Eng. Pract..

[14] Vallejo M., de la Espriella C., Gomez-Santamaria J., Ramirez-Barrera A.F., Delgado-Trejos E. (2019). Soft metrology based on machine learning: A review. Meas. Sci. Technol..

[15] Gryzlov A., Schiferli W., Mudde R. (2016). Soft-sensors: Model-based estimation of inflow in horizontal wells using the extended Kalman filter. Flow Meas. Instrum..

[16] Yang X., Zhang Y., Shardt Y., Li X., Cui J., Tong C. (2020). A KPI-based soft sensor development approach incorporating infrequent, variable time delayed measurements. IEEE Trans. Control Syst. Technol..

[17] Chen J., Lagoa C.M. Observer Design for a Class of Switched Systems. Proceedings of the 44th IEEE Conference on Decision and Control, and the European Control Conference.

[18] Babaali M., Egerstedt M. Asymptotic Observers For Discrete-Time Switched Linear Systems. Proceedings of the IFAC World Congress.

[19] Alessandri A., Coletta P. Design of Observers For Switched Discrete-Time Linear Systems. Proceedings of the American Control Conference.

[20] Koumboulis F.N., Fragkoulis D.G. A Switching Observer Design Scheme for a Double Effect Evaporator. Proceedings of the IEEE International Conference on Industrial Technology (ICIT).

[21] Koumboulis F.N., Fragkoulis D.G. Switching design for the observation of the biomass in Alcoholic Fermentation Processes. Proceedings of the 26th International Conference on Information, Communication and Automation Technologies (ICAT 2017).

[22] Koumboulis F.N., King R.E., Stathaki A. (2007). Logic-based switching controllers—A stepwise safe switching approach. Inf. Sci..

[23] Koumboulis F.N., Tzamzi M.P. Multivariable Step-Wise Safe Switching Controllers. Proceedings of the International Conference on Computational Intelligence for Modelling, Control and Automation and International Conference on Intelligent Agents, Web Technologies and Internet Commerce (CIMCA-IAWTIC’06).

[24] Giannaris G.L., Kouvakas N.D., Koumboulis F.N., Vouyioukas D. (2021). Switching Wireless Control for Longitudinal Quadrotor Maneuvers. J. Intell. Robot. Syst..

[25] Koumboulis F.N., Tzamtzi M.P., Economakos C.E. (2011). Step-wise safe switching control of a constant turning force system. Int. J. Model. Identif. Control.

[26] Eykhoff P. (1974). System Identification Parameter and State Estimation.

[27] Schoukens J., Ljung L. (2019). Nonlinear System Identification: A User-Oriented Road Map. IEEE Control Syst. Mag..

[28] Didi I., Dib H., Cherki B. (2014). An invariant observer for a chemostat model. Automatica.

[29] Guo H., Chen L. (2009). Periodic solution of a chemostat model with Monod growth rate and impulsive state feedback control. J. Theor. Biol..

[30] De Leenheer P., Smith H. (2003). Feedback control for chemostat models. J. Math. Biol..

[31] Dinh M., Fromion V. A RBA model for the chemostat modelling. Proceedings of the 2019 IEEE 58th Conference on Decision and Control (CDC).

[32] Kuenen J.G. (2019). Continuous Cultures (Chemostats). Encyclopedia of Microbiology.

[33] Molin G. (1983). Measurement of the Maximum Specific Growth Rate in Chemostat of Pseudomonas spp. with Different Abilities for Biofilm Formation. Eur. J. Appl. Microbiol. Biotechnol..

[34] Koga S., Humphrey A.E. (1967). Study of the Dynamic Behavior of the Chemostat System. Biotechnol. Bioeng..

[35] Pavlou S. (1987). Dynamics of Chemostat in Which One Microbial Population Grows on Multiple Complementary Nutrients. Biotechnol. Bioeng..

[36] Alcaraz V.G. (2001). Estimation et Commande Robuste Non-Linéaires des Procédés Biologiques de Déppolution des Eaux Usées: Application à la Digestion Anaérobie. Ph.D. Thesis.

[37] Koumboulis F.N., Tzamtzi M.P. A Metaheuristic Approach for Controller Design of Multivariable Processes. Proceedings of the 12th IEEE Conference on Emerging Technologies and Factory Automation.

[38] Koumboulis F.N., Kouvakas N.D. A three term controller for ride comfort improvement. Proceedings of the 19th Mediterranean Conference on Control & Automation.

[39] Åström K.J., Wittenmark B. (2013). Adaptive Control.

[40] Sinha N.K., Rao G.P. (2019). Identification of Continuous-Time Systems: Linear and Robust Parameter Estimation.

[41] Alsuwian T., Amin A.A., Maqsood M.T., Qadir M.B., Almasabi S., Jalalah M. (2022). Advanced Fault-Tolerant Anti-Surge Control System of Centrifugal Compressors for Sensor and Actuator Faults. Sensors.

[42] Fragkoulis D., Li Z., Roux G., Dahhou B. Application of a model based fault isolation method for nonlinear dynamic systems. Proceedings of the 2009 IEEE Conference on Emerging Technologies & Factory Automation.

[43] Paraskevopoulos P.N., Koumboulis F.N. (1992). Observers for singular systems. IEEE Trans. Autom. Control.

[44] Duan G.-R. (2010). Analysis and Design of Descriptor Linear Systems.

[45] Khooban M.-H., Dragicevic T. (2021). Control Strategy for Time-Delay Systems, Part I: Concepts and Theories.

[46] Khooban M.-H., Dragicevic T. (2021). Control Strategy for Time-Delay Systems, Part II: Engineering Applications.

