# Supplementary material for: Soft Sensor Design via Switching Observers
Source: Sensors (Basel). 2023 Feb 13;23(4):2114. doi: 10.3390/s23042114 (PMC9960936; doi:10.3390/s23042114)
Supplement: Supplementary file 1 [file sensors-23-02114-s001.zip › sensors-2172652-supplementary.pdf]

sensors

MDPI

Supplementary Material for

Soft Sensor design via Switching Observers

Fotis N. Koumboulis, Dimitrios G. Fragkoulis, Nikolaos D. Kouvakas and Aikaterini Feidopiasti

Table S1. Scenarios of nominal conditions

Scenario	U [day^{-1}]	X_1 [kg/m^3]	X_2 [kg/m^3]
1	0.1393	0.6502	1.2651
2	0.2293	1.1696	1.1864
3	0.3117	1.7370	1.1004
4	0.3871	2.3571	1.0065
5	0.4584	3.0598	0.9000
6	0.5235	3.8299	0.7833
7	0.5828	4.6741	0.6554
8	0.6369	5.5995	0.5152
9	0.6863	6.6139	0.3615
10	0.7273	7.6154	0.2098

Table S2. Optimal observer parameter selection for $a = 3.16309$

Scenario	$\rho_{F,1}$	$\rho_{F,2}$	$J_{e,A}$	$\tilde{J}_{e,O}$
1	3.16309+	8.11101	0.5	2.26608
2	3.16309+	5.33798	0.5	0.54718
3	3.16309+	4.77700	0.5	0.19104
4	3.16309+	4.57362	0.5	0.07841
5	3.16309+	4.48782	0.5	0.03386
6	3.16309+	4.45649	0.5	0.01506
7	3.16309+	4.45175	0.5	0.00660
8	3.16309+	4.46083	0.5	0.00268
9	3.16309+	4.47730	0.5	0.00090
10	3.16309+	4.49556	0.5	0.00022

Table S3. Optimal observer parameter selection for $a = 4.74463$

Scenario	$\rho_{F,1}$	$\rho_{F,2}$	$J_{e,A}$	$\tilde{J}_{e,O}$
1	4.74463+	5.82279	0.5	2.30452
2	4.74463+	5.38508	0.5	0.57777
3	4.74463+	5.23672	0.5	0.20871
4	4.74463+	5.16769	0.5	0.08826
5	4.74463+	5.13005	0.5	0.03912
6	4.74463+	5.10918	0.5	0.01775
7	4.74463+	5.09744	0.5	0.00788
8	4.74463+	5.09103	0.5	0.00323
9	4.74463+	5.08786	0.5	0.00109
10	4.74463+	5.08679	0.5	0.00027

Table S4. Minimum and maximum metric values for $a = 3.16309$

Scenario	Minimum $J_{e,A}$	Maximum $J_{e,A}$	Minimum $\tilde{J}_{e,O}$	Maximum $\tilde{J}_{e,O}$
1	0.4452	0.5	2.26608	2.2968
2	0.2899	0.5	0.54718	0.6001
3	0.2317	0.5	0.19104	0.2255
4	0.2032	0.5	0.07841	0.0988
5	0.1870	0.5	0.03386	0.0453
6	0.1775	0.5	0.01506	0.0212
7	0.1716	0.5	0.00660	0.0097
8	0.1680	0.5	0.00268	0.0041
9	0.1657	0.5	0.00090	0.0014
10	0.1644	0.5	0.00022	0.00035

Table S5. Minimum and maximum metric values for $a = 4.74463$

Scenario	Minimum $J_{e,A}$	Maximum $J_{e,A}$	Minimum $\tilde{J}_{e,O}$	Maximum $\tilde{J}_{e,O}$
1	0.1747	0.5	2.30452	2.4033
2	0.1102	0.5	0.57777	0.6290
3	0.0850	0.5	0.20871	0.2367
4	0.0721	0.5	0.08826	0.1041
5	0.0643	0.5	0.03912	0.0479
6	0.0595	0.5	0.01775	0.0225
7	0.0563	0.5	0.00788	0.0103
8	0.0541	0.5	0.00323	0.0043
9	0.0526	0.5	0.00109	0.0015
10	0.0516	0.5	0.00027	0.00037

Table S6. Identification process results

Scenario	h_D	$\hat{h}_D$	Fluctuation	h_N	$\hat{h}_N$	Fluctuation
1	1.5815	1.55785	11.23%	8.3499	8.2062	10.61%
2	1.2422	1.21168	2.66%	7.8307	7.5833	2.74%
3	0.9648	0.95675	1.94%	7.2631	7.20247	1.86%
4	0.7390	0.735441	1.57%	6.6428	6.61295	1.46%
5	0.5500	0.548011	1.30%	5.9403	5.92086	1.42%
6	0.3983	0.397059	0.99%	5.1695	5.15708	1.22%
7	0.2774	0.276654	1.00%	4.3259	4.31716	1.03%
8	0.1816	1.180927	1.31%	3.4013	3.39306	1.01%
9	0.1060	0.105466	2.04%	2.3868	2.3823	1.34%
10	0.0521	0.0516387	3.61%	1.3839	1.37502	1.84%

Table S7. Identification error norms and steady state estimation error

Scenario	$J_{e,O}^{*}/u_w^2$ ($a=3.16309$)	$J_{e,O}^{*}/u_w^2$ ($a=4.74463$)
1	2.11381	2.14967
2	0.533495	0.563329
3	0.187899	0.205286
4	0.0778057	0.0875864
5	0.0336782	0.0389032
6	0.0150149	0.0176936
7	0.00658277	0.00786332
8	0.00267343	0.00321651
9	0.000898796	0.00108247
10	0.000219048	0.000262766

Table S8. Observer parameter and target operating areas

Scenario	U_i	$\rho_{F,1}$	$\rho_{F,2}$	$U_i - u_{i,\max}$	$U_i + u_{i,\max}$
1	0.1393	3.5+	6.7289	0.1183	0.1603
2	0.2293	3.5+	5.1269	0.1869	0.2718
3	0.3117	3.5+	4.7294	0.2401	0.3833
4	0.3871	3.5+	4.5732	0.2757	0.4985
5	0.4584	3.5+	4.5018	0.2894	0.6274
6	0.5235	3.5+	4.4716	0.2707	0.7742
7	0.5828	3.5+	4.4621	0.2013	0.7742
8	0.6369	3.5+	4.4637	0.0384	0.7742
9	0.6863	3.5+	4.4716	0	0.7742
10	0.7273	3.5+	4.4817	0	0.7742

Table S9. Observer parameter and target operating areas

Area	U_i	h_D	h_N	$\hat{h}_D$	$\hat{h}_N$	$\rho_{F,1}$	$\rho_{F,2}$	$U_i - u_{i,\max}$	$U_i + u_{i,\max}$
1	0.065	1.8904	8.7165	1.8479	8.4729	3.5+	488.6804	0.0566	0.0734
2	0.080	1.8259	8.6464	1.7883	8.4360	3.5+	18.4288	0.0694	0.0906
3	0.105	1.7209	8.5253	1.6952	8.3719	3.5+	9.2990	0.09029	0.1197
4	0.140	1.5788	8.3462	1.5515	8.1911	3.5+	6.7010	0.1189	0.1611
5	0.190	1.3857	8.0688	1.3698	7.9680	3.5+	5.5310	0.1579	0.2221
6	0.280	1.0678	7.4935	1.0614	7.4450	3.5+	4.8404	0.2210	0.3390
7	0.520	0.4059	5.2147	0.4046	55.2005	3.5+	4.4726	0.2728	0.7672

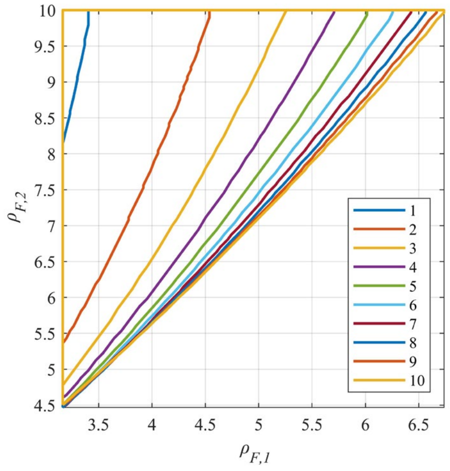

Figure S1. Acceptable observer poles' regions for $a = 3.16309$ and all scenarios of nominal points

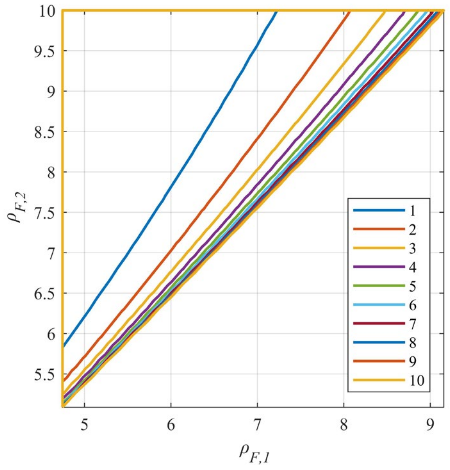

Figure S2. Acceptable observer poles' regions for $a = 4.74463$ and all scenarios of nominal points

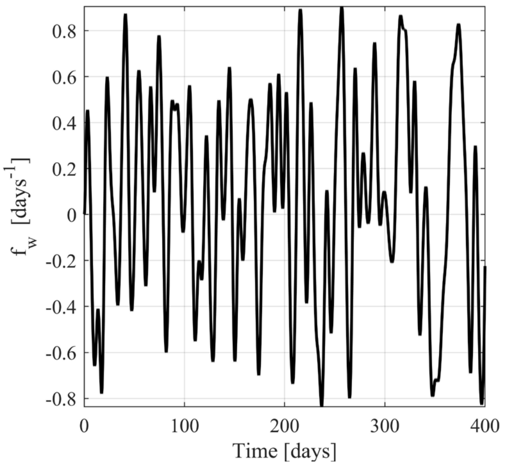

Figure S3. Actuation signal $f_w(t)$

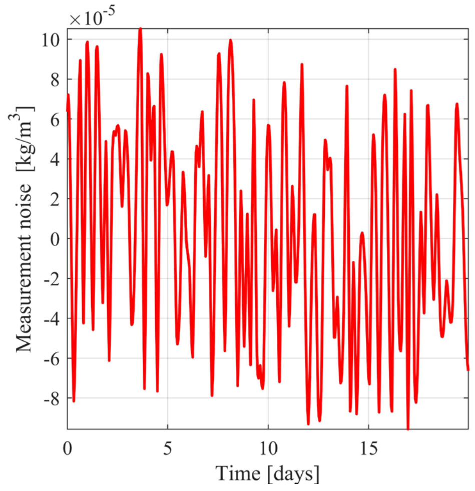

Figure S4. Additive measurement noise

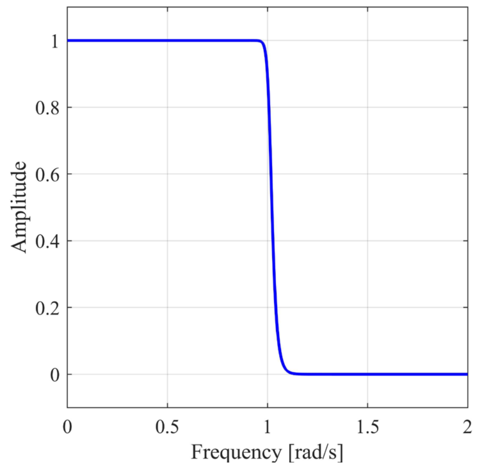

Figure S5. Butterworth filter Bode plot

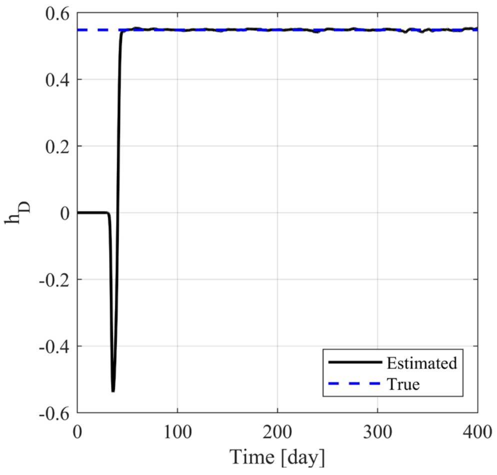

Figure S6. Estimation of h_D for scenario 5.

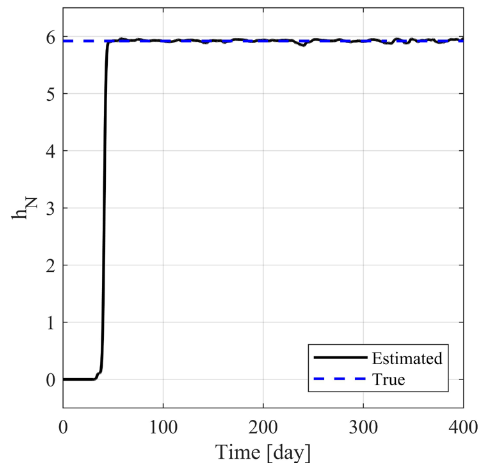

Figure S7. Estimation of h_N for scenario 5.

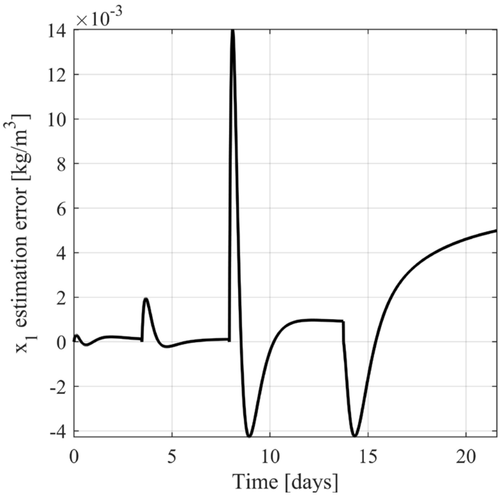

Figure S8. Substrate concentration estimation error.

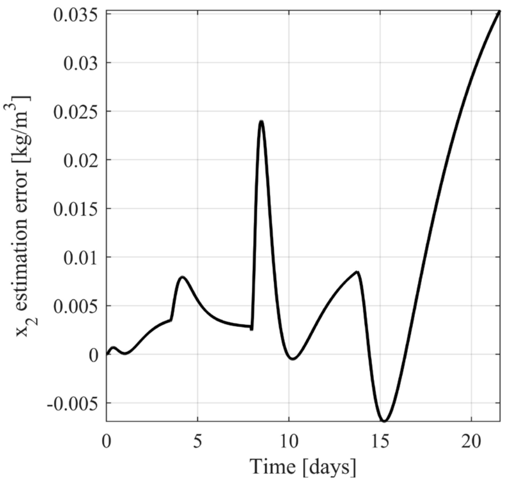

Figure S9. Microorganism concentration estimation error.

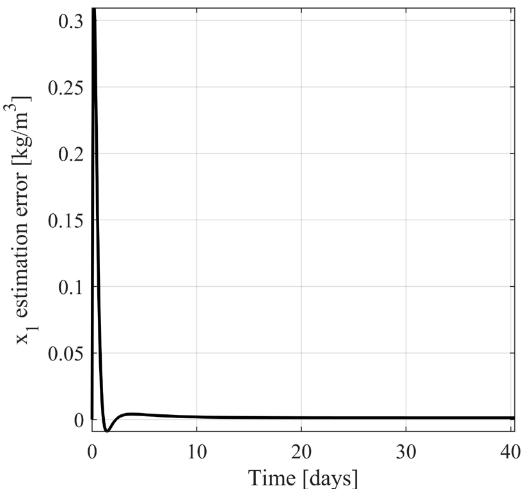

Figure S10. Substrate concentration estimation error for single step transition.

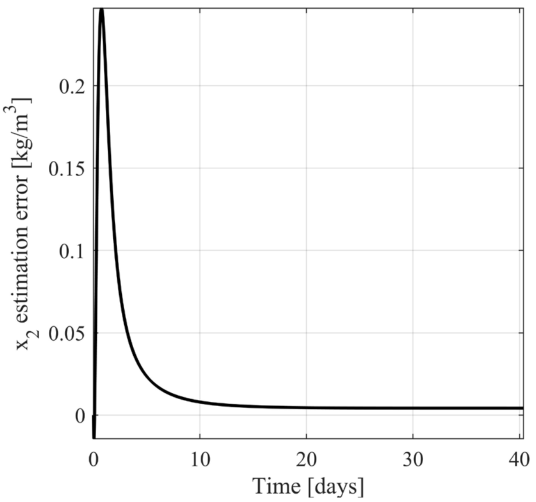

Figure S11. Microorganism concentration estimation error for single step transition.

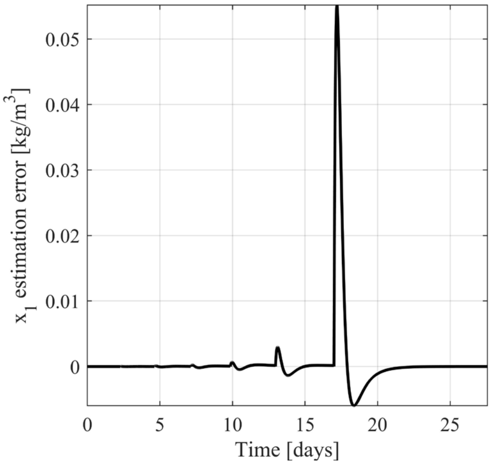

Figure S12. Substrate concentration estimation error.

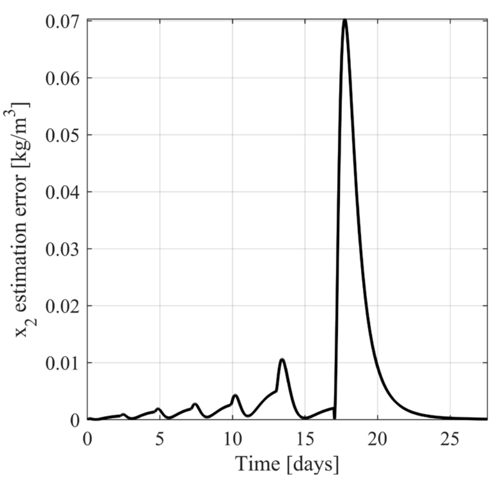

Figure S13. Microorganism concentration estimation error.
